# Whole genome sequencing distinguishes skin colonizing from infection-associated *Cutibacterium acnes* isolates

**DOI:** 10.3389/fcimb.2024.1433783

**Published:** 2024-10-24

**Authors:** Andreas Podbielski, Thomas Köller, Philipp Warnke, Israel Barrantes, Bernd Kreikemeyer

**Affiliations:** ^1^ Institute of Medical Microbiology, Virology and Hygiene, Rostock University Medical Center, Rostock, Germany; ^2^ Research Group Translational Bioinformatics, Institute for Biostatistics and Informatics in Medicine and Aging Research, Rostock University Medical Center, Rostock, Germany

**Keywords:** *Cutibacterium* (*Propionibacterium*) *acnes*, GWAS - genome-wide association study, pangenome analyses, SLST, SNP

## Abstract

**Introduction:**

*Cutibacterium acnes* can both be a helpful colonizer of the human skin as well as the causative agent of acne and purulent infections. Until today, it is a moot point whether there are *C. acnes* strains exclusively devoted to be part of the skin microbiome and others, that carry special features enabling them to cause disease. So far, the search for the molecular background of such diverse behavior has led to inconsistent results.

**Methods:**

In the present study, we prospectively collected *C. acnes* strains from 27 infected persons and 18 healthy controls employing rigid selection criteria to ensure their role as infectious agent or colonizer. The genome sequences from these strains were obtained and carefully controlled for quality.

**Results:**

Deduced traditional phylotyping assigned almost all superficial isolates to type IA1, while the clinical strains were evenly distributed between types IA1, IB, and II. Single locus sequence typing (SLST) showed a predominance of A1 type for the control strains, whereas 56% of the clinical isolates belonged to types A1, H1 and K8. Pangenome analysis from all the present strains and 30 published genomes indicated the presence of an open pangenome. Except for three isolates, the colonizing strains clustered in clades separate from the majority of clinical strains, while 4 clinical strains clustered with the control strains. Identical results were obtained by a single nucleotide polymorphism (SNP) analysis. However, there were no significant differences in virulence gene contents in both groups.

**Discussion:**

Genome-wide association studies (GWAS) from both the pangenome and SNP data consistently showed genomic differences between both groups located in metabolic pathway and DNA repair genes. Thus, the different behavior of colonizing and infectious C. acnes strains could be due to special metabolic capacities or flexibilities rather than specific virulence traits

## Introduction

*Cutibacterium acnes* is a Gram-positive, facultative anaerobic rod that is a dominant component of the skin microbiome of healthy humans (Bay et al., 2020; Skowron et al., 2021). For its role as a commensal, the bacterium has been known for decades (Achermann et al., 2014), although there have been several changes of its taxonomic association from *Bacillus* to *Corynebacterium*, *Propionibacterium* and finally *Cutibacterium acnes* (Scholz and Kilian, 2016). Simultaneous to the most recent taxonomic change of genus, three subspecies – *Cutibacterium acnes* ssp. *acnes*, *defendens*, and *elongatum* – have been described by different groups (McDowell et al., 2016; Dekio et al., 2015, 2019). Based on rDNA, *hly* hemolysin and *recA* DNA repair gene sequences as well as multi-locus typing schemes, phylotypes I (i.e. IA_1_, IA_2_, IB, IC), II, and III are discriminated, which correspond to the three subspecies (McDowell et al., 2005, 2008, 2012a; Kwon et al., 2013; Achermann et al., 2014).

Starting with its first identification, a pathogenic role for *Cutibacterium acnes* in acne vulgaris has been assumed, which is reflected by its species name. However, a causative role in systemic infections was a moot point until recently – three studies on the significance of *C. acnes* in blood cultures exclusively assigned them a status as contaminants (Park et al., 2011; Tenderenda et al., 2022; Weinstein et al., 1997). Since then, *C. acnes* has been associated with catheter-related bloodstream infection (Badia-Cebada et al., 2022; Boman et al., 2022), infective endocarditis primarily associated with prosthetic valves and cardiac devices (Alonso-Menchén et al., 2024; Banzon et al., 2017; Lindell et al., 2018; Sohail et al., 2009), prosthetic joint infections (Abad et al., 2019; Auñón et al., 2022; Sanderson et al., 2022) especially in shoulder arthroplasty (Jacquot et al., 2023; Kurihara et al., 2023; Torrens et al., 2024), breast-implants (Hanna et al., 2023) and contact-lens associated keratitis or postoperative endophthalmitis (Ashby et al., 2023; Fowler et al., 2021).

Most probably, *C. acnes* has host-beneficial and -detrimental roles (Brüggemann et al., 2021; Boyanova, 2023) due to its involvement in metabolism of skin fatty acids (Skowron et al., 2021) as well as in the balance of local defense mechanisms (Dréno et al., 2020) and simultaneously, production of virulence factors (listed e.g. by Achermann et al., 2014; Brüggemann et al., 2021; Erbežnik et al., 2023; Yu et al., 2024). An example for the dual role of assumed pathogenetic traits is the potential for biofilm formation: strains displaying a prominent biofilm formation were found to be associated with a superficial habitat in healthy persons, acne vulgaris as well as chronic and severe clinical infections (Achermann et al., 2014; Brüggemann et al., 2021; Cavallo et al., 2022; Coenye et al., 2021; Hsu et al., 2022; Kuehnast et al., 2018; Nakase et al., 2021).

Similarly important are its interactions with other components of the skin microbiome (Ahle et al., 2022; Rozas et al., 2021; Szabó et al., 2023). So according to current ideas on the pathogenesis of acne, dysbiosis of the skin microbiome including shifts in its own strain diversity is the major trigger factor for development and persistence of acne and atopic dermatitis (Blicharz et al., 2021; Cavallo et al., 2022; Chan et al., 2023; Ferček et al., 2021; Yu et al., 2024), which in turn opens therapeutic options by probiotic manipulations (Polak et al., 2021). Skin dysbiosis could even have impact on the infection pathogenesis in remote anatomical compartments such as bones and joints (Chen et al., 2021) as in turn, remote microbiomes could influence skin conditions (De Pessemier et al., 2021; Han et al., 2021; Sánchez-Pellicer et al., 2022).

Another not competitive but rather additive explanation to a selective role of *C. acnes* in atopic skin diseases is a differing contribution by the diverse *C. acnes* phylotypes or subspecies (Cavallo et al., 2022; Corvec et al., 2019; McLaughlin et al., 2019; Pécastaings et al., 2018; Spittaels et al., 2020). So a dysbalance between the phylotypes could lead to unusual amounts of metabolic products such as porphyrins which could activate the inflammasomes of keratinocytes (Dagnelie et al., 2019; Spittaels et al., 2021) or L-carnosine which as an antioxidative stress metabolite attenuates skin inflammation (Yu et al., 2024).

A similar divergence of *C. acnes* phylotypes/subspecies has been observed in deep seated infections (Lee et al., 2020; McDowell et al., 2012b; Ponraj et al., 2023; Salar-Vidal et al., 2021). However, because of the ubiquitous presence of *C. acnes* on the skin, there is always the possibility that skin isolates could contaminate specimens from inner anatomical compartments (generally considered as sterile) during sampling and laboratory processing and therefore, influence studies on specific differences between skin and deep seated infection isolates. Several groups have addressed this problem and have suggested measures or algorithms by which *C. acnes* contaminants can be discriminated from true infectious isolates (Boman et al., 2022; Otto-Lambertz et al., 2024; Park et al., 2011; Weinstein et al., 1997).

In parallel, several groups have established more refined typing schemes for *C. acnes* including whole genome sequencing (WGS) approaches, which could potentially reveal relevant pathotypes among *C. acnes* and its subspecies (Cobian et al., 2021, Erbežnik et al., 2023, Ponraj et al., 2022; Salar-Vidal et al., 2021; Scholz et al., 2014; Torrens et al., 2022; Yu et al., 2024).

In the present study, we analyze molecular differences between *C. acnes* strains prospectively collected from the skin of clinical healthy persons and from patients with deep seated and potentially detrimental infections. Therefore, we sought to combine protocols ensuring the clinical relevance of *C. acnes* strains and the most refined technique for molecular analyses, i.e. WGS. Using this approach, we could show a clear cut difference between the isolates from healthy and infected persons demonstrated by the mutually exclusive presence of single nucleotide polymorphisms (SNPs) within several housekeeping genes of the two entities.

## Materials and methods

### Study protocol

Two distinct sets of *C. acnes* isolates were prospectively collected in the period January to October 2019.

One set included strains obtained from 18 healthy volunteers working at the University Hospital Rostock. None of them worked in close vicinity to patients but instead in offices, transport and laboratories. None of them suffered from acne during the sampling period or reported to have suffered from it in their teenage and adolescent days.

A single swab sample was taken from the supranasal forehead of each volunteer at the beginning of their individual daily shifts. Inclusion criteria were the personal statement of a currently healthy status, and the absence of underlying chronic diseases.

The other set included 27 patient strains isolated from invasively gained samples. The patients were located at the clinics of surgery and internal medicine as well as at the anesthesiologic intensive care unit. One inclusion criterion was an age above 18 years. Furthermore, only patients were included from which at least two samples from a specific anatomical site were obtained and both of which containing only *C. acnes* but no other bacteria or fungi. From these, only the positive sample arriving first at the laboratory was included in the analysis. Unlike the strict criteria expressed by Boman et al. (2022), clinical data were not used as an inclusion criterion, since the data were not available for all patients. In any case, no information on present or former acne periods accompanied the patient samples. Given the age of the patients however, acne was an improbable diagnosis for all but the 18 year old female (v04016).

### Handling of clinical and volunteers’ samples

The samples were handled according to the standard operation procedures of the fully Deutsche Akkreditierungsstelle (DAkkS)-accredited laboratory based on DIN EN ISO 15189.

In detail, the swabs were cultured on Schaedler agar enriched with 5% sheep blood (BectonDickinson, Heidelberg, Germany) for 120 h at 36 ± 1°C under anaerobic conditions (80% N_2_, 10% H_2_, 10% CO_2_) obtained by the Anoxomat III device (Advanced Instruments, Norwood, MA, USA) in appropriate jars. After inspecting the plates at 72 and 120 h incubation, potential *C. acnes* colonies were passaged on the same medium and then subjected to MALDI-TOF analysis using the MALDI Biotyper sirius IVD System (Bruker Daltonics, Bremen, Germany). Measurements were carried out according to the IVD-MALDI Biotyper standard procedure protocol with the MBT Compass IVD Software (v.4.3) utilizing the MBT IVD Library (v.11, revision G, 2021, Bruker Daltonics).

Strains identified as *C. acnes* were subjected to antibiotic resistance testing by using E test gradient strips (bioMérieux, Marcy L’Etoile, France) and the Fastidious Anaerobe Agar with horse blood (ThermoFisher Scientific, Bremen, Germany) according to the European Committee on Antimicrobial Susceptibility Testing (EUCAST) guidelines v.12.0 (http://www.eucast.org). Thus, MIC breakpoints for benzylpenicillin, meropenem, vancomycin and clindamycin were set at 0.06, 0.125, 2, and 0.25 mg/l. For tetracycline there are no breakpoints defined by EUCAST. Therefore, the E test results are shown without interpretation.

All *C. acnes* strains were stored in Microbank™ Blue Colour Beads and Cap storage vials (Pro-Lab, Richmond Hill, Canada) at -80°C until further analysis.

### DNA sequencing

Total chromosomal and plasmid DNA from all *C. acnes* strains was prepared within one week by using a NUCLISENS**^®^** easyMAG**^®^** nucleic acid extraction system (bioMérieux, Nürtingen, Germany GmbH) according to the manufacturer’s recommended protocol. The quantity and purity of the extracted DNA were determined using a NanoDrop™ 2000 spectrophotometer (Thermo-Fisher Scientific, Waltham, MA, USA).

The extracted total DNAs from all *C. acnes* strains were subjected to next-generation sequencing (NGS)-based assessment of whole genomes in 2 individual 600-cycle sequencing runs (14 clinical isolates, 9 control strains and one negative control each per run).

For this, 1 ng of purified DNA from all isolates was used to prepare individual libraries employing the Illumina Nextera^©^ XT DNA Library Prep Kit (Illumina, San Diego, CA, USA) according to the manufacturer’s instructions. An Agilent Technology 2100 Bioanalyzer (Agilent Technologies, Santa Clara, CA, USA) served to verify fragmentation and also final library fragment size distribution on a High Sensitivity DNA Chip. AMPure XP beads (Beckman Coulter GmbH, Krefeld, Germany) were used for DNA library purification.

The 2 final pooled libraries were applied to MiSeq Reagent v3 600-cycle Kits and sequenced on a MiSeq system as two 300-cycle paired-end runs. 10% PhiX control library was spiked into each final library pool. A cluster density of 1248 (K/mm2) and 1577 (K/mm2) was achieved with 87.3% of clusters passing filter specifications during run 1 and 2, respectively.

During run 1, roughly 32.9 million reads of 37.7 million total reads passed filter specifications. Metrics of run 2 revealed 30.9 million reads of 35.3 million total reads passed filter specifications. The Q30 score reached an average of 55% in both runs. Index reads were quite evenly distributed across all *C. acnes* samples, reaching 13 to 18% of total reads per sample in each run. In both runs only 0.1% of the total reads matched to the negative control samples. In both runs 94.9% and 92.7% of the passed filter reads were identified as *C. acnes* associated, respectively.

### Bioinformatics

Adapters were removed from the paired end sequencings with Scythe version 0.991 (Buffalo, 2011), and trimmed with Sickle version 1.33 (Joshi and Fass, 2011). Trimmed sequences were then filtered for the PhiX phage, via mapping against the phage PhiX sequence with Bowtie2 version 2.4.4, and recovering the unmapped fragments (Langmead and Salzberg, 2012).

Then the obtained filtered sequences were assembled using multiple *k*-mer sets with SPAdes version 3.15.3 (Bankevich et al., 2012). Assembly scaffolds were then annotated with Prokka version 1.14.6 (Seemann, 2014). Further annotation was achieved with antiSMASH, a program to find gene clusters encoding secondary metabolites (Blin et al., 2021).

Genome and proteome completeness was assessed through the search for known orthologous genes (COGs) present in the Propionibacteriales group with the BUSCO program version 5.2.2 (Simao et al., 2015). For this and further comparisons, we retrieved the complete *C. acnes* reference genomes from GenBank.

Typing was carried out with the Single-locus sequence typing (SLST) database (Scholz et al., 2014; retrieved on 2022-08-19) through blastn searches with identity and e-value cutoffs of 100% and 0.0 respectively (version 2.12.0+; Camacho et al., 2009), and the identification of phages was performed with Phaster (Accessed 2022-02-28; Arndt et al., 2016) and for finding plasmids we used blastn searches against the NCBI UniVec database.

Then we built a pangenome (core and accessory genes) from the encoded proteins in both the *C. acnes* reference and the novel isolate genomes, with the program Roary version 3.13.0 (Page et al., 2015). Afterwards we searched for SNPs within the novel isolates with respect to the reference genomes with snippy version 4.6.0 (Seemann, 2020), a tool that minimizes false positives in variant calling (Bush, 2021). Variant calling allows identification of SNPs and small insertions and deletion (indels) from NGS data. These SNPs were then employed to reveal recombination sites, via the Gubbins program version 2.4.1 (Croucher et al., 2015).

Finally, both the pangenome and the variant data were used in genome- wide association study (GWAS) experiments with the Scoary tool version 1.6.16 (Brynildsrud et al., 2016). For this, the genome coordinates matching with phage and recombinations were previously excluded from the SNP results, and the pangenome predictions were directly used in Scoary. All sequencings from the novel isolates were deposited in the European Nucleotide Archive (ENA) database under the study accession number PRJEB52145. A summary of the bioinformatic procedures is shown in the **Supplementary Figure 1**.

### Ethical considerations

From the volunteers, a written consent for study participation was obtained prior to the sampling procedure. Patients gave an informed consent for usage of the samples for study purposes as a part of the treatment contract signed upon hospital admission.

Beginning with the -80°C storage process, all person-related data except for gender, age and type of specimen (healthy control vs. blood culture, bone/joint, deep tissue samples) was deleted to ensure a maximum of anonymity.

The study was approved by the Rostock University research ethics committee (registration number A2023-0074).

## Results

### Volunteers’ (healthy controls) and patients’ data, *C. acnes* antibiotic resistance phenotypes

The volunteers’ age ranged between 34 and 67 years (arithmetic mean 48.4 years, median 47.5 years). Thirteen persons were females, five males (**Table 1**). All *C. acnes* strains isolated from this group were susceptible to penicillin, meropenem, clindamycin, and vancomycin.

**Table 1:** Origin, SLST-/traditional phylo-typing results of control and clinical strains.

isolate designation	gender	age [years]	ward within hospital	SLST type	traditional phylotype	sample type
	of donor/patient					
D13	f	34	n.a.	A1	IA_1_	superficial/healthy control
D14	f	62	n.a.	A1	IA_1_	superficial/healthy control
D15	f	56	n.a.	A1	IA_1_	superficial/healthy control
D16	m	67	n.a.	A1	IA_1_	superficial/healthy control
D17	f	48	n.a.	A1	IA_1_	superficial/healthy control
D18	f	50	n.a.	A1	IA_1_	superficial/healthy control
D19	f	40	n.a.	A1	IA_1_	superficial/healthy control
D20	f	63	n.a.	A1	IA_1_	superficial/healthy control
D21	f	55	n.a.	K2	II	superficial/healthy control
K1	f	44	n.a.	A1	IA_1_	superficial/healthy control
K2	m	44	n.a.	A1	IA_1_	superficial/healthy control
K5	f	34	n.a.	A1	IA_1_	superficial/healthy control
K7	f	50	n.a.	A25	IA_1_	superficial/healthy control
K8	m	35	n.a.	D1	IA_1_	superficial/healthy control
K9	m	39	n.a.	A1	IA_1_	superficial/healthy control
K10	m	44	n.a.	A1	IA_1_	superficial/healthy control
K11	f	59	n.a.	C1	IA_1_	superficial/healthy control
K12	f	47	n.a.	A1	IA_1_	superficial/healthy control
b02908	m	71	Intern.	K7	II	systemic/blood culture
b03014	m	68	Surgery	H1	IB	deep seated tissue
b03270	m	80	Intern.	K1	II	systemic/blood culture
b04239	m	53	Intern.	H4	IB	systemic/blood culture
b04269	m	64	Intern.	K1	II	systemic/blood culture
b04543	m	85	Intern.	H1	IB	systemic/blood culture
b04764	f	61	ICU	A1	IA_1_	systemic/blood culture
b05051	m	67	ICU	H4	IB	systemic/blood culture
b05914	m	74	Intern.	H1	IB	systemic/blood culture
b05979	m	60	ICU	K7	II	systemic/blood culture
v01026	m	73	Surgery	H1	IB	deep seated tissue
v03925	f	27	Surgery	K8	II	deep seated tissue
v03992	m	85	surgery	A1	IA_1_	deep seated tissue
v04016	f	18	Surgery	A1	IA_1_	deep seated tissue
v04083	m	44	Surgery	A1	IA_1_	bone/joint
v04290	m	81	Intern.	K8	II	bone/joint
v04857	m	43	Intern.	K8	II	deep seated tissue
v04915	m	73	Surgery	H1	II	bone/joint
v06486	m	73	Surgery	D1	IA_1_	deep seated tissue
v07195	f	63	Surgery	D1	IA_1_	deep seated tissue
v07786	m	29	Surgery	A5	IA_1_	deep seated tissue
V07926	m	77	Surgery	excluded from further analysis		deep seated tissue
v07956	m	71	Surgery	K7	II	deep seated tissue
v08288	m	46	Surgery	H1	IB	deep seated tissue
v08359	f	23	Surgery	K8	II	deep seated tissue
v12811	m	35	Surgery	C1	IA_1_	bone/joint
v14082	m	83	Surgery	H1	IB	bone/joint

The genomes from the novel isolates were used for similarity searches against the SLST database (Scholz et al., 2014; retrieved on 2022-08-19) with blastn version 2.12.0+, with identity and e-value cutoffs of 100% and 0.0 respectively. Only the highest scoring fragments (according to the blastn bitscore values) were selected for typing.

n.a., not applicable.

The patients’ age ranged between 18 and 85 years (arithmetic mean 60.3 years, median 67.5 years). Five patients were females, twenty-two males. For further details of patient data, refer to **Table 1**. All *C. acnes* strains isolated from this group were susceptible to penicillin, meropenem, and vancomycin. With respect to clindamycin, only isolates v03925 and v07786 displayed resistance to this compound. Deduced from WGS results, a mutation in the 23S rDNA gene was responsible for that phenotype in both cases. Since tetracycline is commonly used for *C. acnes* skin infections, the strains were tested even without a defined EUCAST breakpoint. All isolates displayed tetracycline MIC values of <0,25 mg/l.

### Sequencing and typing of the novel *C. acnes* genomic isolates

Sequencing of the 45 isolates resulted in 64.55 million Illumina reads (8.56 Gb), with an average of 1.4 million reads (188.61 Mb) per isolate. These Illumina reads were then assembled individually, for a total of 114.99 million assembled bases, with genomes sizes ranging from 2.48 to 2.56 Mb (2.51 Mb average), coverage from 44.55 to 292.34x, and encoding an average of 2,319 proteins (minimum 2,291; maximum 2,389 open reading frames; **Supplementary Table 1**). The largest assembled contigs varied from 154.47 to 893.64 kb, with an average %GC of 59.37 (**Supplementary Table 1**).

The genome completeness was assessed by comparing the proteomes from novel isolates against the proteomes from all existing complete genomes from *C. acnes* present in GenBank (**Supplementary Table 2**), and using as a reference the proteins present in the Propionibacteriales datasets from the BUSCO software (Simao et al., 2015). From these analyses we found that the analyzed genomes miss 1-2% of the genes, except for those from the strains SK137 (GenBank accession NC_014039) and C1 (accession NC_018707), which lack 6.9 and 7% of these genes respectively (**Supplementary Figure 2**).

Afterwards, and to study the relationships between the novel sequences of these isolates, we first classified their genome sequences based on their SLST patterns (**Table 1**). We found that the isolates pertained to eleven SLST types, predominantly types A1 (40%), H1 (18%) and K8 (9%). Between the sample types, H1 was the most abundant both in the bone and joint (40%) and deep tissue samples, while A1 was more commonly identified between the superficial anatomic sites/healthy controls (78%), and the types H1, H4, K1 and K7 were evenly distributed between the systemic isolates (22% each).

When translating the SLST results into the traditional phylotyping scheme, all but one strain (phylotype II) from healthy controls belonged to phylotype IA_1_, while the clinical strains were almost evenly distributed between phylotypes IA_1_ (8 isolates), IB (10 isolates), and II (9 isolates). Phylotypes IC and III associated with SLST types G1 and L1, respectively, were not identified in any of the analyzed *C. acnes* strains (**Table 1**).

In addition, we further annotated these novel genomes for phage sequences (**Supplementary Table 3**). All identified phages were predicted as incomplete prophages (hit genes count = 6), and typically being associated with Corynebacteria, either to the Adelaide (six isolates) or the Lederberg (7 genomes) types. Regardless of the clinical sample type (superficial/deep seated/systemic), both Adelaide or Lederberg prophages were identified in single *C. acnes* genomes, except for two genomes sequenced from bone/joints strains that contained exclusively Adelaide prophages (**Supplementary Table 3**).

Based on blastn searches against the NCBI UniVec database no linear plasmid as described by Salar-Vidal et al. (2021) was detected in any strain.

### The *C. acnes* genomes from novel isolates

Afterwards, we carried out a pangenome analysis, including the novel isolates and all known complete *C. acnes* genomes (**Supplementary Table 2**), to study the gene diversity of these novel isolates in the context of this species. In this manner, genes were clustered as follows: (i) core genes (present in 99–100% of the genomes: 1,600 genes); (ii) shell genes (15–99% of the genomes: 1,246 genes); and (iii) cloud genes (present in less than 15% of the genomes: 1,199 genes).

A comparison of the number of genomes versus the gene content in the pangenome showed that the number of conserved genes decreased while the total number of genes in the pangenome increased as genomes are incorporated into the analysis (**Supplementary Figure 3**).

**Figure 1:**
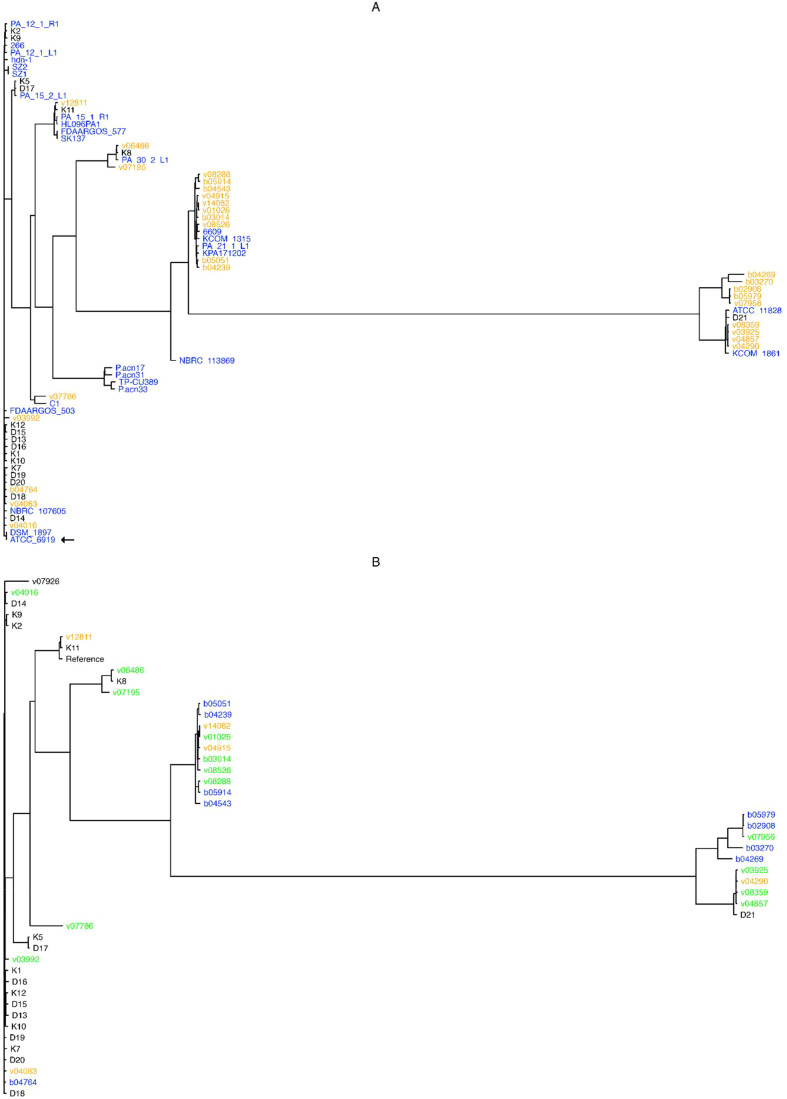
Pangenome- and SNP- based cladograms. Trees were built with FastTree (Price et al., 2010), with the GTR substitution model for nucleotide sequences (-gtr -nt) from the alignment of core pangenome genes **(A)**, or from SNPs obtained upon the alignment of all novel isolates against the NCBI GenBank designated *C. acnes* reference genome HL096PA1. **(B)**. Colors in **(A)** stand for genome isolate groups, e.g. orange for novel genomes isolated from bone and joint, deep seated tissues or systemic, black for superficial anatomical novel isolates, and blue for known complete *C. acnes* genomes; while in **(B)** colors stand for bone and joint (orange), systemic (blue), or deep tissue (green) isolates, and black for those isolates that could not be classified as any from the former categories. The type strain ATCC 6919 is indicated by an arrow.

These results indicate that *C. acnes* possesses an open pangenome, and hence that the addition of newly sequenced isolates helps indeed in the characterization of these bacteria at the genomic level. In addition, a phylogenetic analyses based on the alignment of all 1,600 core genes revealed that the studied *C. acnes* strains and isolates can be divided into 13 distinct clades (**Figure 1A**). The cladogram shows that most of the “orange” novel isolates (19 genomes isolated from bone/joint samples, deep seated tissues or blood cultures; 70% of all novel isolates) clustered into two major clades: One, featuring a “black” genome (superficial isolate D21), and two complete known genomes, namely ATCC 11828 (Horvath et al., 2012; originally isolated from a subcutaneous abscess), and KCOM 1861, which was obtained from a jaw osteomyelitis lesion (Park et al., 2011); and another, that entailed four known complete genomes: 6609, KCOM 1315, PA_21_1_L1, and KPA171202.

Briefly, almost all superficial (K and D) isolates clustered outside the clades from the clinical (B and V) isolates, except for the isolates K8 and D21 that clustered between the systemic and deep seated tissue samples; while the isolates v03992, v04016, v04083, and b04764 were not located within the clades with the most systemic and deep seated tissue samples (**Figure 1A**).

### Recombination and single nucleotide polymorphisms in the novel isolates

To study the hotspots for recombinations and SNPs, we first masked all previously identified phage regions in all novel isolate genomes (**Supplementary Table 3**) with the maskfasta function of BEDtools (Quinlan and Hall, 2010), and then aligned all these masked genomes against the NCBI GenBank designated reference genome *C. acnes* HL096PA1. The alignment was then cleaned for zero or low coverage regions as well as for poor quality genotype regions with snippy (Seemann, 2020), and the output (clean alignment) was then used to identify common recombination sites in the novel genomes with the Gubbins program (Croucher et al., 2015). This resulted in 373 unique recombination sites from 1,490 unique annotated loci, as compared to the NCBI GenBank designated reference genome *C. acnes* HL096PA1 (total 3,413 pangenome loci with recombinations matching the reference genome; **Supplementary Data Sheet 1**). Afterwards, functional annotation of genes within recombination hotspots was performed to identify potential processes associated with regions prone to genetic exchange.

In this manner, we observed seven major recombination regions with over 500 SNPs, entailing 278 genes; 29 of these genes fully annotated and with known functions (**Supplementary Table 4** and **Supplementary Figure 4**). The annotated functions comprised genes involved in the protein synthesis: ribosomal proteins (*rpsI, rplM, rplQ, rpsD, rpsK, rpsM, rpmJ*), tRNA modification genes (*trmB, truA*), the *infA* translation initiation factor IF-1, the preprotein translocase *secY*, and the *map* type I methionyl aminopeptidase genes; as well as Cytochrome-c-oxidase (COX) subunits (*ctaD, coxB*), excinuclease ABC subunits (*uvrB, uvrA*), fatty acid biosynthesis (*fabG, fabI*) genes, among others.

The highest number of recombinations was found in the region of the genes alanine racemase, *alr*, and class II fumarate hydratase gene *fumC*, involved in the conversion of L-alanine and D-alanine and the TCA cycle respectively (**Supplementary Figure 4**). In addition, an antiSMASH search (Blin et al., 2021) revealed that the *uvrB* gene is within a RiPP-like cluster (formerly annotated as bacteriocin by antiSMASH), i.e. a region encoding unspecified ribosomally synthesized and post-translationally modified peptide product (RiPP) genes (**Supplementary Table 5**).

Then, we studied the phylogenetic relationships between the isolates from the perspective of the SNP data. For this, we first masked the recombinant regions previously found by the Gubbins program from the NCBI GenBank designated reference genome *C. acnes* HL096PA1 with BEDtools (Quinlan and Hall, 2010), and then aligned the novel genomes (previously masked for phage regions) against it, to find highly polymorphic SNP positions that were independent of the recombination and phage insertion events.

We found 293 core SNPs (average 68.26 SNPs per novel isolate), that individually ranged from 2 (isolate K11) to 198 (isolate b05979) SNPs with respect to the NCBI GenBank designated reference genome *C. acnes* HL096PA1. The rate of transitions versus transversions went from 2 (isolate v12811) to 14 (isolate v07786), with the K11 genome displaying no transversions. No multiallelic variants or indels were identified.

Besides, we found 15 chromosomal regions with over 5 SNPs per 0.1 kb (**Supplementary Table 6**), corresponding to 12 unique loci. These loci entailed a single noncoding region and 11 protein coding genes; the latter corresponding to subunits of the excinuclease ABC (UvrABC), cell cycle proteins (FtsX-like, FtsW), a uridylyltransferase (locus PAGK_RS02615), the adenosylcobinamide-phosphate synthase CbiB (also known as CobD), a cobyric acid synthase (locus PAGK_RS02250), the Cytochrome-c-oxidase (COX) subunit 4 (locus PAGK_RS07450), two loci from the zinc ABC transporter substrate-binding protein, an RNA-binding protein (locus PAGK_RS11720), and a DUF6350 family protein (locus PAGK_RS08770; **Supplementary Table 6**).

Finally, the multiple genome alignment cleaned for zero or low coverage regions as well as for poor quality genotype regions was used to build a cladogram, to study the evolutionary relationships between these novel *C. acnes* isolates, with respect to the NCBI GenBank designated reference genome *C. acnes* HL096PA1. For this, we used the FastTree program (Price et al., 2010) with the generalized time reversible (-gtr) substitution model applied to nucleotide sequences (-nt).

The resulting tree shows that isolates pertaining to the systemic (b) and deep seated tissue (v) groups clustered together, except for the v03992, v04016, v04083, and b04764 genomes; conversely, most superficial isolates (K and D genomes) were found outside the systemic and deep tissue clades, except for the genomes K11, K8 and D21 (**Figure 1B**).

### Loci associated to clinical phenotypes from the novel isolates

In order to search for loci linked to clinical phenotypes from the novel isolates, we performed GWAS analyses both from the pangenome and SNP data with the Scoary program (Brynildsrud et al., 2016). For this, and in order to have at least 10 isolates per group, all deep tissue, bone/joint, and systemic isolates were clustered into a single category called “clinical isolates”, while the remaining were classified as “healthy controls”.

**Figure 2:**
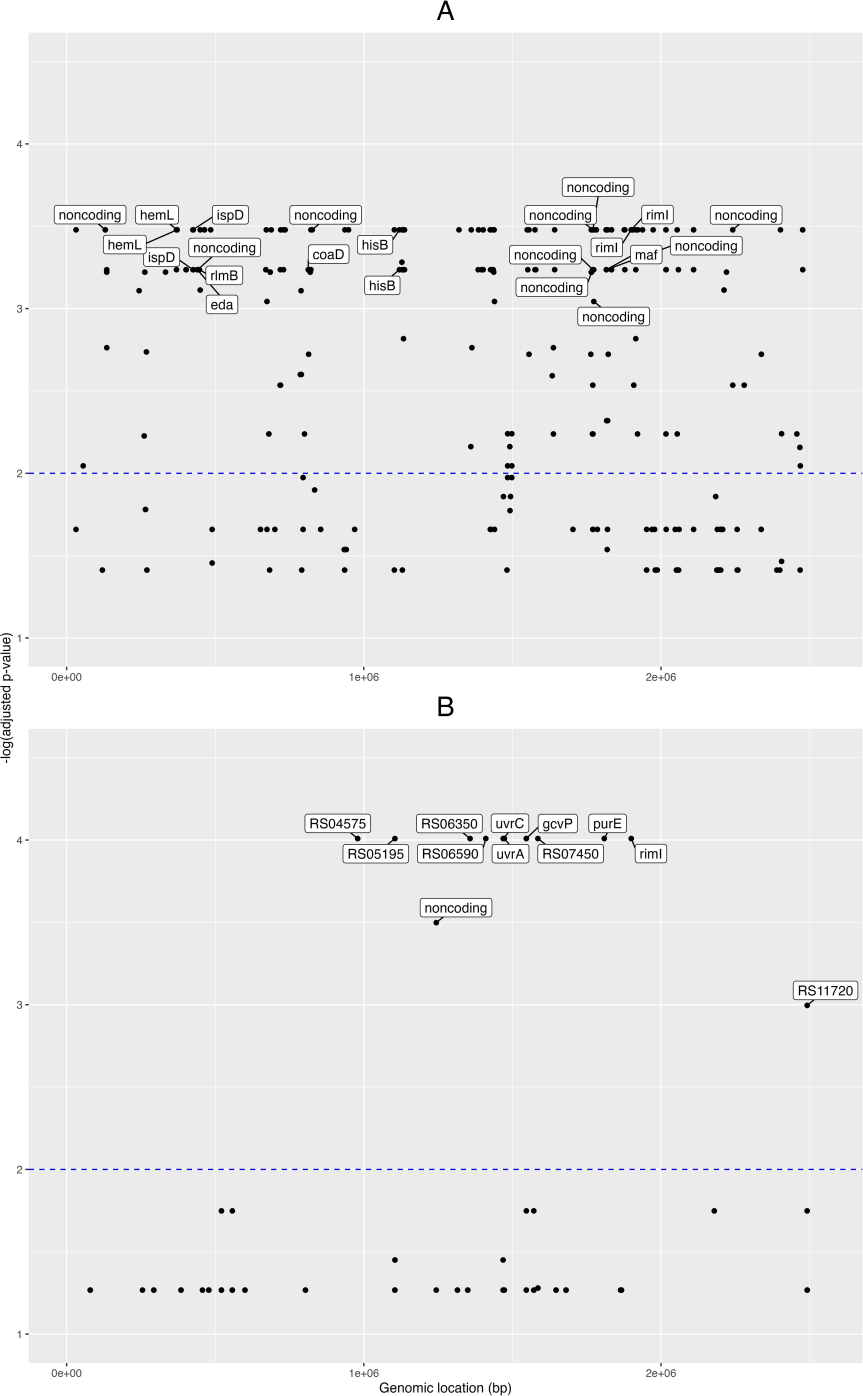
Pangenome- and SNP- based genome wide associations. Manhattan plots from the genome-wide associations (GWAS) for clinical phenotypes in the novel *C. acnes* isolates. GWAS for **(A)** pangenome data, and **(B)** single nucleotide polymorphisms (SNPs). Labels for the pangenes **(A)** are shown for those with -log(adjusted p-value) > 3 and with loci fully annotated, while labels for the genes corresponding to SNPs **(B)** are shown for those with -log(adjusted p-value) > 2. The x-axis shows the genomic positions (in base pairs) for the pangenes and SNPs along the reference genome; and the y-axis is the minus logarithm for the association (adjusted *p*-value).

First, for the pangenome- based GWAS analysis, we employed the gene presence/absence table generated by the Roary program (Page et al., 2015) during the pangenome analysis, and this was used as input for Scoary together with the table of traits (isolate vs. condition, e.g. “clinical” or “healthy”). In this manner we obtained 388 pangenomic fragments associated with clinical traits (Benjamini-Hochberg adjusted p-value < 0.05; **Supplementary Data Sheet 2**). Then we obtained the nucleotide sequences from these 388 pangenome loci and mapped these onto the NCBI GenBank designated reference genome *C. acnes* HL096PA1, to see the distribution of these loci across the genome. A plot of the matching pangenomic loci associated to the “clinical” traits, together with their corresponding GWAS adjusted p-values, can be seen in the **Figure 2A**.

Eight of these loci were fully annotated genes (adjusted p-value < 0.001): the *coaD* pantetheine-phosphate adenylyltransferase, the *eda* bifunctional aldolase, *hemL* aminomutase, *hisB* imidazoleglycerol-phosphate dehydratase, *ispD* cytidylyltransferase, *maf* nucleoside triphosphate pyrophosphatase, *rimI* ribosomal protein S18-alanine N-acetyltransferase, and the *rlmB* 23S rRNA (guanosine(2251)-2’-O)-methyltransferase.

Then, for the GWAS analysis based on the SNP data, we took the nucleotide polymorphisms previously found, filtered for phage and recombination sites, and used it as input for Scoary together with the trait metadata. Here, we found 52 SNP positions pertaining to 15 loci (adjusted p-value < 0.05; **Supplementary Data Sheet 3**); and from these 15 loci, six were fully annotated genes: the *gcvP* glycine dehydrogenase, *iolB* 5-deoxy-glucuronate isomerase, *purE* imidazole ribonucleotide mutase, *rimI* ribosomal protein S18-alanine N-acetyltransferase, and the excinuclease ABC subunits *uvrA* and *uvrC* (**Figure 2B**).

When comparing the novel isolates versus NCBI GenBank designated reference genome *C. acnes* HL096PA1, we found 4 SNPs in *rimI*; three between the genomic segments 1,899,800 and 1,899,900, and one between 1,899,900 and 1,900,000. Finally, to understand better the relationships between the RimI orthologues encoded in the studied genomes, we aligned the encoded protein sequences and built a phylogenetic tree (**Supplementary Figure 5**). This cladogram shows that all healthy control isolates except D21 clustered on one main branch together with 8 of 27 patient isolates. D21 is the only phylotype II isolate among the healthy control strains, while the patient strains on this branch comprise all isolates belonging to phylotype IA_1_. Thus the detected RimI sequence differences discriminate phylotype IA_1_ strains from other phylotypes.

## Discussion

In contrast to several former studies, in this study we used i) two, ii) prospectively gathered *C. acnes* strain collections, both with iii) a well-defined clinical, i.e. healthy or infected background. In order to stress the etiological relevance in the collection of strains from deep seated or severe infections, iv) only isolates were accepted when *C. acnes* was diagnosed from at least two samples of the respective anatomical compartment as the v) single identified pathogen. All the strains collected under these prerequisites vi) were subjected to a WGS approach to examine the genomes for the relatedness of their pan- and accessory genomes and the presence of recombinations and SNPs. Finally, we performed GWAS analyses to test for the presence or absence of specific genes in the genomes of the two groups.

In order to increase the discriminatory power, we analyzed vii) only one isolate from each sample, although we were aware that two or more *C. acnes* strains of different SLST- or even phylotypes can be present at a given healthy or infected anatomical site (Scholz et al., 2014; El Sayed et al., 2019; Ponraj et al., 2022; Yu et al., 2024). We were also aware that the exclusion of samples with the simultaneous presence of Cutibacteria and other bacterial species increases the probability of a causative role of *C. acnes* in infected sites (Otto-Lambertz et al., 2024), but clearly is not a definitive criteria for the etiology of the infection. Simultaneously, truly pathogenic strains in mixed infections could be missed by such a rigid standard.

In the group of clinical cases, male gender predominated, which is in line with results from previous studies (Boman et al., 2022; Otto-Lambertz et al., 2024). The same applies to the aspect of age – the chance of comorbidities as an established risk factor for *C. acnes* infections (Otto-Lambertz et al., 2024) increases in elderly persons, and consistently the arithmetic mean and median of our patients was well above 60 years. Due to their role as hospital employees, in the group of healthy volunteers female gender predominated (**Table 1**). The volunteers were also younger than the patients, although old enough to avoid phases of rapid changes in strain compositions seen in maturing adults (Hu et al., 2023). Except for one patient, all persons included in the study were well beyond the typical age for acne vulgaris.

Regarding the traditional phylotypes, the overwhelming majority of skin isolates from healthy volunteers belonged to type IA_1_, while the clinical isolates were quite evenly distributed between types IA_1_, IB, and II (**Table 1**). This is in line with previous results (Dekio et al., 2019; Scholz et al., 2014 for superficial isolates; Erbežnik et al., 2023; Lee et al., 2020; Kurihara et al., 2023; Salar-Vidal et al., 2021 for clinical isolates). As described by Ponraj et al. (2022) for implant-associated and Kurihara et al. (2023) for surgery-associated isolates without demonstrating the exclusive presence of potential virulence genes in these strains (Cobian et al., 2021), the three most frequent SLTS types of the present clinical strains - collected from a variety of severe infections - belonged to the H-, K- and A1-types.

Since even refined typing schemes based on single or few genetic loci did not reveal solid and reproducible differences between colonizing and infecting *C. acnes* strains in the literature, the WGS approach was chosen in the present study. When comparing the obtained genome sequences with those from data collections, the average genome size and gene content (**Supplementary Table 1**) was well in line with published data (2.49 Mb, 2,319 open reading frames [ORFs] vs. 2.51 Mb, 2,331 ORFs) (Cobian et al., 2021; Yu et al., 2024). Since we and others (Cobian et al., 2021) described an open pangenome, genome size and gene content variation in selected strains could be a consequence thereof.

As stated for general genome size and gene content, also the content and type of phages, antibiotic resistance genes and the vast majority of documented and potential virulence genes (such as the CAMP factor, DeoR porphyrin synthesis repressor, GehAB lipases, HtaA iron acquisition protein, Hyl hyaluronate lyase, NarH nitrogen turnover, SigH sigma factor, SodA superoxide dismutase, SrtF sortase, RoxP radical oxygenase, Tly hemolysin, and TufA elongation factor) did not vary between the colonizing and infecting strains (**Supplementary Tables 3**, **5**), an observation that is in line with previous publications (Brüggemann et al., 2021; Cobian et al., 2021; Erbežnik et al., 2023; Kurihara et al., 2023; Yu et al., 2024).

A different picture prevails once the pangenomes are compared (**Figure 1A**). In this approach, with the exception of three isolates, the strains isolated from superficial sites clustered outside the clades containing the majority of clinical strains. The only phylotype II isolate (D21) was among the three separately clustering strains. The analysis of the SNP-phylogeny led to an identical result (**Figure 1B**). In turn, the pangenome comparison clustered the clinical isolates into 5 clades. One containing 4 isolates from blood, bone/joint, and deep seated tissue was identical with the one containing the majority of healthy control strains. These 4 isolates belonged to phylotype IA_1_. Although the SNP-phylogeny assigned the clinical isolates to 6 clades, again the four IA1 isolates were the only ones that were found in the now 2 clades containing the majority of the control isolates. Altogether, this could indicate that the four clinical strains were contaminants in spite of our selection mode. As expected, WGS allowed a much finer discrimination of *C. acnes* strains than any of the traditional typing methods. However, irrespective of the mode of analysis of the WGS data the clustering of the clinical strains was not strictly associated with the patients’ infection type.

When applying a genome-wide recombination analysis and, simultaneously, a genome-wide SNP analysis on every genome sequence included in this study, only a few hotspots were identified. The recombination hotspots were located in noncoding regions and besides, in housekeeping genes involved in protein synthesis, DNA repair and the TCA cycle (**Supplementary Tables 4**, **5**). The SNP hotspots were almost exclusively located within coding regions, similarly affecting housekeeping genes and genes with ill-defined but probably regulatory functions (**Supplementary Table 6**).

When focusing on the isolates of the present study and subjecting their pangenomes and SNPs to GWAS analyses (**Figures 2A, B**), in both cases sequence differences only in housekeeping genes were detected between the colonizing and all infecting strains. Yet, the results from both approaches did not point to an identical set of housekeeping genes, but in both cases several of the genes were involved in energy metabolism. This finding - partially - contradicts the results from Erbežnik et al. (2023) and Kim et al. (2023), which found small if at all differences in metabolic genes and pathways between the *C. acnes* phylotypes. It is in line with the results of Cobian et al. (2021), who described very few housekeeping genes that were specific for the genomes of phylotypes IC and IIA.

In a recent extremely thorough analysis, Yu et al. (2024) compared genome sequences obtained from 1,234 *C. acnes* isolates retrieved form 11 healthy persons as well as 11 and 10 patients with atopic dermatitis and moderate acne, respectively. Although addressing a different study population, these authors could show that the genetic heterogeneity of *C. acnes* is influenced both by individual and skin site again in both normal and diseased skin. In addition, the heterogeneity is most probably a consequence of horizontal gene transfer and selection pressure, leading to more genes related to energy production and conversion, amino acid transport and metabolism, translation, ribosomal structure and biogenesis and inorganic ion transport and metabolism in acne-associated strains.

Interestingly, in our study, both the pangenome- and SNP- based GWAS analyses indicated that the *rimI* gene, encoding a ribosomal protein S18-alanine N-acetyltransferase, is linked to clinical traits displayed by the isolates, as indicated by a significant pangenome locus (adjusted p-value < 0.00033) and two highly significant SNPs (adjusted p-value < 9.7979e-05).

The RimI acetyltransferase was originally described as the enzyme responsible of the N- terminal acetylation of the S18 ribosomal protein (Yoshikawa et al., 1987). The *rimI* gene is well conserved among Gram-negative and Gram-positive bacteria. Later experiments showed that RimI is able to modify the elongation factor Tu in *E. coli*, and hence is involved in the regulation of the protein synthesis in this organism (Pletnev et al., 2022). Knock down mutation of *rimI* in *E. coli* does not affect growth, while overexpression enhances growth (Lee and Back, 2023). The RimI protein from *Mycobacterium tuberculosis* displays a relaxed substrate specificity, i.e. it is able to acetylate proteins other than its original target, the S18 ribosomal protein (Pathak et al., 2016), and thus is candidate to regulate bacterial processes other than those first described for this enzyme. RimI has also been shown to confer resistance to peptide antibiotics and quinolones (Huynh et al., 2023; Kazakov et al., 2014). Finally, as certain bacterial virulence factors are acetylated, it has been suggested that protein acetylation might be linked to bacterial virulence (Ren et al., 2017). Taken together, our results with this cohort of novel genomic isolates suggest an association between allelic variants of the *C. acnes rimI* gene and the clinical traits, and this association might be due to its impact on multiple bacterial processes by RimI, such as the regulation of the protein synthesis and the bacterial virulence.

The present study has several limitations. First, the cohort size of the patient group is relatively small, thus a correlation between type of infection and genome data could have been missed as well as strains from healthy control persons could be more diverse than in the present collective.

Second, the gender and age distribution in the two groups of healthy and diseased persons differs due to the fact that the healthy control persons were recruited from the hospital staff. Thus, the identified differences in *C. acnes* genomes from both groups must be confirmed by testing more elder males. However, when testing elder persons, the chance of finding healthy volunteers decreases.

Third, only one strain per person was analyzed although the simultaneous presence of two or even several phylo-/SLST- types has been described in clinical samples. Thus, a potential variation in genome content could have been missed with respect to infection entity and anatomical site.

Fourth, the antibiotic treatment of the patients was not recorded in the case of the infection-associated strains, while such a treatment could have a selective effect on the diagnostic culture results. Yet, this drawback is common to most if not all other studies on the genome data from clinical *C. acnes* isolates.

Fifth, the etiological relevance of the clinical strains is not proven, in spite of the selection criteria we used when collecting the strains. The consistent clustering of four clinical strains with the control strains as well of three control strains with the clinical isolates in different genome analyses points into this direction. However the consistent separate clustering of the other clinical and control strains indicates their status as colonizing or infectious strains.

In spite of these limitations, we were able to characterize the novel isolates at the clinical and genomic level to an extent that allowed to identify significant differences between the control and infection-associated strains.

## Conclusion

In the present study, we used carefully selected *C. acnes* strains from healthy control persons and infected patients to identify molecular differences between the two entities. The majority of strains from both groups clustered in different clades, indicating the presence of discernible genomic variations. These could not be correlated with the presence or absence of known or putative virulence genes, but rather with the absence or presence of housekeeping genes or at least, an increased occurrence of recombinations and SNPs in such genes. This situation resembles the differences that discriminate acne-supporting phylotype IA strains from such strains of the same phylotype, which are not associated to this disease. Therefore, we suggest to invest more time and effort to elucidate whether metabolic fitness and adaptation rather than classical pathogenic principles could be the relevant difference between colonizing and infectious *C. acnes* strains.

## Data Availability

The datasets presented in this study can be found in online repositories. The names of the repository/repositories and accession number(s) can be found below: https://www.ebi.ac.uk/ena, PRJEB52145.

## Supplementary material

Supplementary Figure 1 Bioinformatic methods. Overview of the bioinformatic methods applied to analyze the novel isolates genomes, from their assembly, annotation and individual typing (SLST, antibiotic resistance, phages), to the comparisons versus the complete genomes (pangenome analysis, variant calling, recombinations), including SNP- and pangenome- based GWAS.

Supplementary Figure 2 Genome completeness. Graphic display of the sequence coverage obtained for the novel *C. acnes* genomes. Complete, fragmented and - compared to the reference genome – missing sequence ratios are shown by color codes. Genomes corresponding to the type strain ATCC 6919 are indicated with arrows.

Supplementary Figure 3 Gene content in the pangenome. Pangenome plot of *C. acnes* across the genomes used for pangenome construction. The number of genomes included for pangenome construction are shown in the x-axis, while the y-axis indicated the number of identified genes.

Supplementary Figure 4 Genome-wide recombinations. Genome- wide map of recombinations in *C. acnes* based on the reference genome. The density (SNPs per segment) is shown for recombination blocks in the novel isolate genomes overlapping protein coding regions based on the NCBI GenBank designated reference genome HL096PA1. An arbitrary cutoff of 500 SNPs per segment was used to highlight the coding regions with the highest number of recombinations.

Supplementary Figure 5 Cladogram of the *rimI* orthologs. The tree was built with FastTree (Price et al., 2010) from the alignment of *rimI* orthologs encoded in their corresponding pangenome genes. Colors follow the convention of the **Figure 1B**, i.e. bone and joint (orange), systemic (blue), or deep tissue (green) isolates, and black for those isolates that could not be classified as any from the former categories. The genome corresponding to the type strain ATCC 6919 is indicated with an arrow.

Supplementary Table 1 Sequencing summary. Results from the overall bioinformatic analyses of the novel *C. acnes* sequences are shown in this table. Sequencing/Reads: overall no. of reads obtained from the specific *C. acnes* genome; bases: overall no. of bases read. Assembly/Length: length of the assembled genome in megabases; largest contig: size of the largest contig obtained from the genome of a specific strain, N50: median size of the contigs obtained from this strain; coverage: fold-coverage of the overall read sequence with respect to the specific genome. Annotation: coding sequences (CDS) annotated to the specific overall sequences; no. of ribosomal (tRNA), transfer (tRNA) and transfer messenger (tmRNA) RNA CDS detected in the specific sequence. The three rRNAs listed correspond to single copies of the 5S, 16S and 23S rRNA genes, except for D16 and b04239, that possess extra and incomplete copies of the 16S and 23S rRNA genes, respectively.

Supplementary Table 2 Complete *C. acnes* genomes from RefSeq. Complete *C. acnes* genomes included in the study. The respective sizes, strain names and references are indicated for each NCBI RefSeq accession. Genomes corresponding to the type strain (NZ_CP023676 and NZ_CP044255) are indicated in bold. The type strain is stored in different databases and repositories and therefore has several unique descriptors. Isolation source is indicated if retrievable from primary and secondary references. ^1^ Search in primary and secondary reference unfortunately did not allow easy grouping into acne and healthy individual isolates since tables showing isolate names and isolation sources for these isolates are missing in those publications.

Supplementary Table 3 Phage predictions. Prophages were identified with PHASTER (Arndt et al., 2016). “Most common” stands for the most commonly found phage by PHASTER, and “Accession” is the NCBI genome accession for this most commonly found phage.

Supplementary Table 4 Recombination sites. Recombination sites were identified with Gibbons upon comparison of the complete *C. acnes* genomes versus the NCBI GenBank designated reference genome HL096PA1. “Start” and “end” indicate the genomic coordinates with respect to the HL096PA1 genome (as well as their annotations in “gene” and “description”), while “value” is the density (SNPs per segment) to the corresponding genome position. Due to use of genomic coordinates instead of whole gene segments, the alr, coxB, erpA, and fumC genes appear more than once, i.e. these genes entail more than one recombination site.

Supplementary Table 5 Biosynthetic gene clusters. Listed are genes pertaining to biosynthetic gene clusters identified with antiSMASH in the *C. acnes* NCBI GenBank designated reference genome HL096PA1, together with their annotations (gene locus, product, function). These genes map to either the LAP (Linear azoline- containing peptides) or RiPP (unspecified ribosomally synthesized and post-translationally modified peptide product)- like regions, that both produce post-translationally modified peptides with various biological activities.

Supplementary Table 6 Regions with high SNP density. Single nucleotide polymorphisms (SNPs) were identified with snippy (REF) upon comparison of the novel infection-associated isolates versus the complete *C. acnes* genomes versus the NCBI GenBank designated reference genome HL096PA1. “Start” and “end” indicate the genomic coordinates with respect to the HL096PA1 genome (as well as their annotations in “gene” and “description”), while “value” is the density (SNPs per 0.1 Kb) to the corresponding genome position.

Supplementary Data Sheet 1 Recombination sites.

Supplementary Data Sheet 2 GWAS from pangenome data.

Supplementary Data Sheet 3 GWAS from SNP data.
