# Supplementary figures and images for: Whole genome sequencing distinguishes skin colonizing from infection-associated Cutibacterium acnes isolates
Source: Front Cell Infect Microbiol. 2024 Oct 24;14:1433783. doi: 10.3389/fcimb.2024.1433783 (PMC11540793; doi:10.3389/fcimb.2024.1433783)

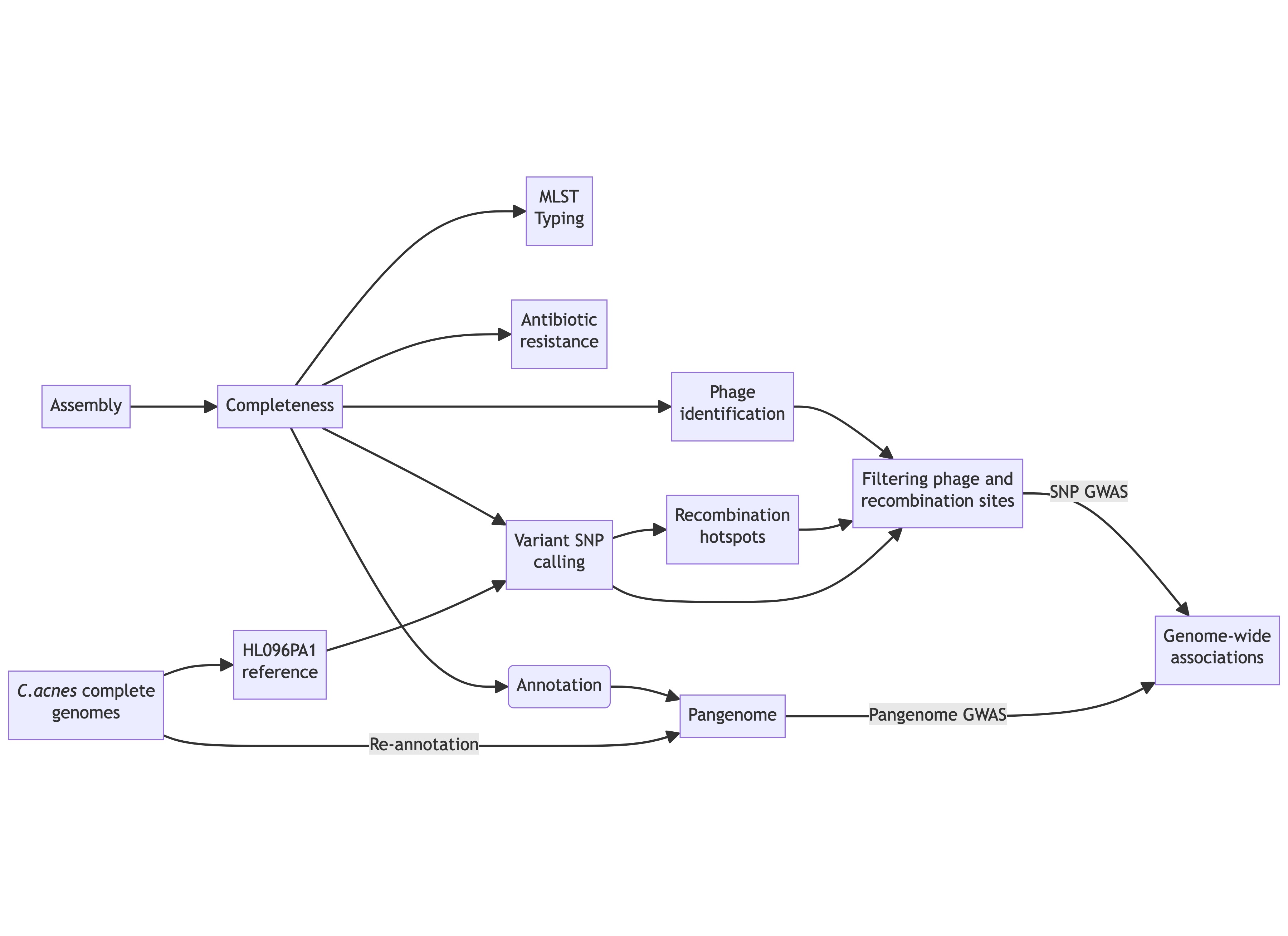

Supplement: Supplementary Figure 1 — Bioinformatic methods. Overview of the bioinformatic methods applied to analyze the novel isolates genomes, from their assembly, annotation and individual typing (SLST, antibiotic resistance, phages), to the comparisons versus the complete genomes (pangenome analysis, variant calling, recombinations), including SNP- and pangenome- based GWAS. [file Image1.jpg]

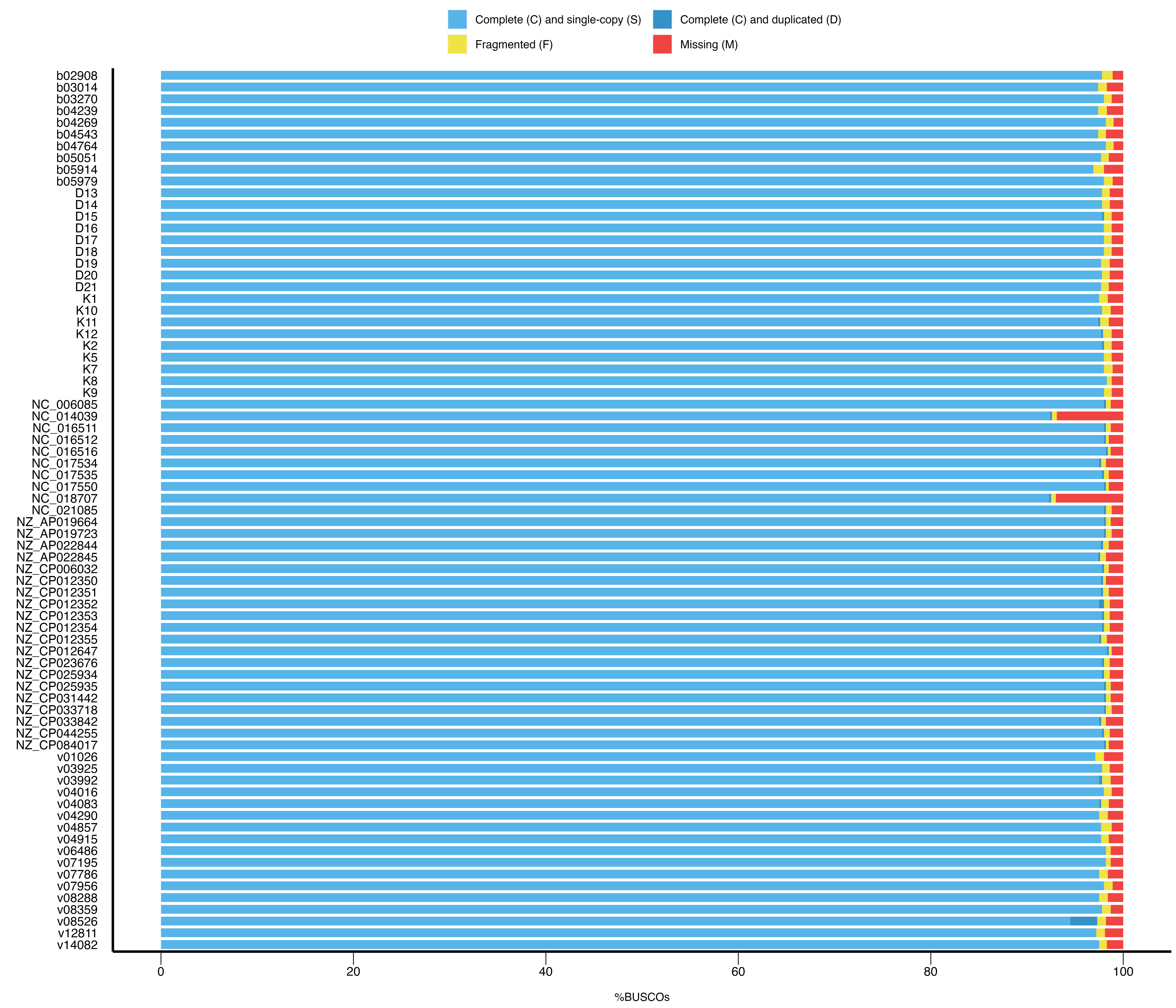

Supplement: Supplementary Figure 2 — Genome completeness. Graphic display of the sequence coverage obtained for the novel C. acnes genomes. Complete, fragmented and - compared to the reference genome – missing sequence ratios are shown by color codes. Genomes corresponding to the type strain ATCC 6919 are indicated with arrows. [file Image2.jpg]

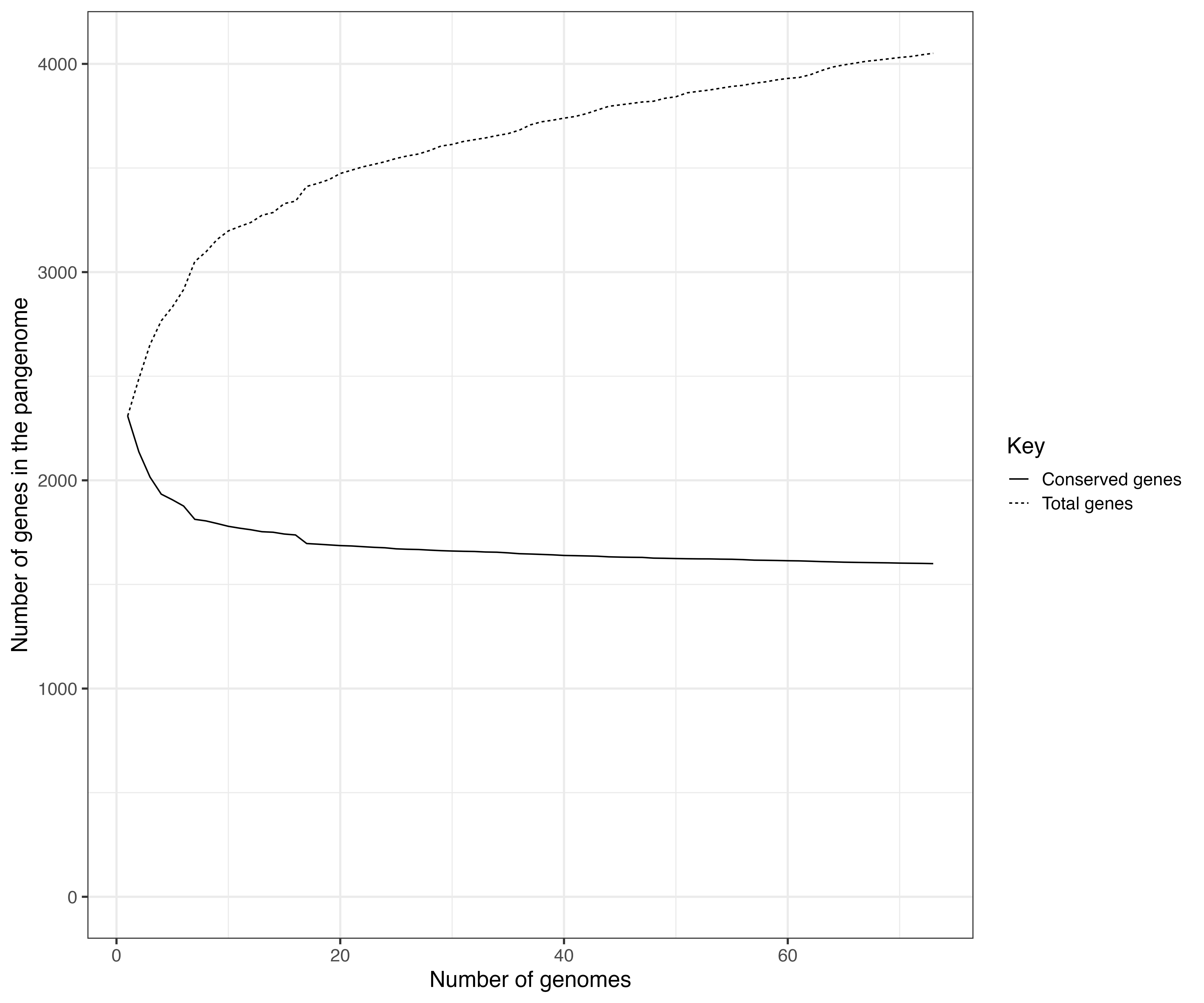

Supplement: Supplementary Figure 3 — Gene content in the pangenome. Pangenome plot of C. acnes across the genomes used for pangenome construction. The number of genomes included for pangenome construction are shown in the x-axis, while the y-axis indicated the number of identified genes. [file Image3.jpg]

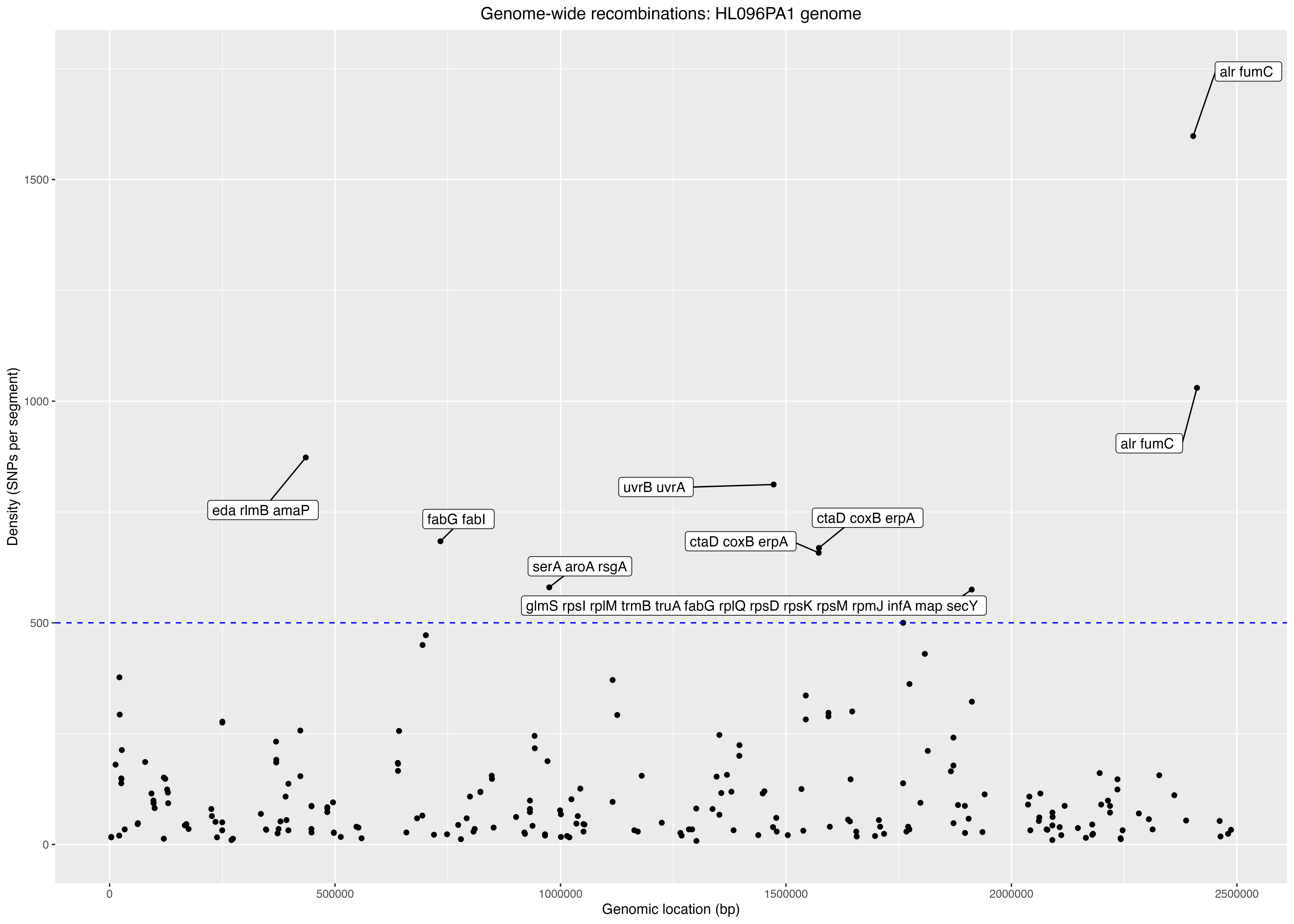

Supplement: Supplementary Figure 4 — Genome-wide recombinations. Genome- wide map of recombinations in C. acnes based on the reference genome. The density (SNPs per segment) is shown for recombination blocks in the novel isolate genomes overlapping protein coding regions based on the NCBI GenBank designated reference genome HL096PA1. An arbitrary cutoff of 500 SNPs per segment was used to highlight the coding regions with the highest number of recombinations. [file Image4.jpg]

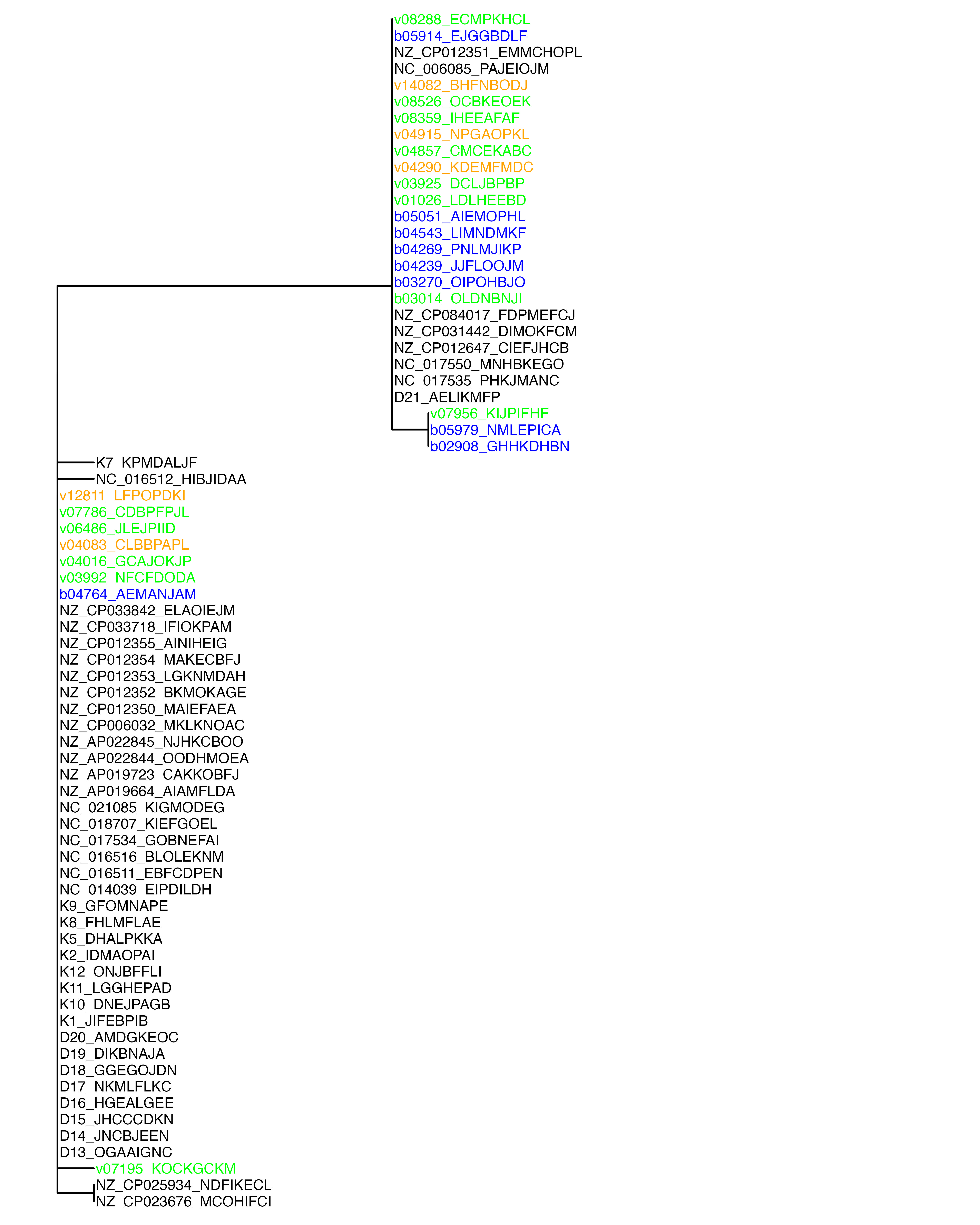

Supplement: Supplementary Figure 5 — Cladogram of the rimI orthologs. The tree was built with FastTree (Price et al., 2010) from the alignment of rimI orthologs encoded in their corresponding pangenome genes. Colors follow the convention of the Figure 1B , i.e. bone and joint (orange), systemic (blue), or deep tissue (green) isolates, and black for those isolates that could not be classified as any from the former categories. The genome corresponding to the type strain ATCC 6919 is indicated with an arrow. [file Image5.jpg]
