# Supplementary material for: Whole genome sequencing distinguishes skin colonizing from infection-associated Cutibacterium acnes isolates
Source: Front Cell Infect Microbiol. 2024 Oct 24;14:1433783. doi: 10.3389/fcimb.2024.1433783 (PMC11540793; doi:10.3389/fcimb.2024.1433783)
Supplement: Supplementary Table 1 — Sequencing summary. Results from the overall bioinformatic analyses of the novel C. acnes sequences are shown in this table. Sequencing/Reads: overall no. of reads obtained from the specific C. acnes genome; bases: overall no. of bases read. Assembly/Length: length of the assembled genome in megabases; largest contig: size of the largest contig obtained from the genome of a specific strain, N50: median size of the contigs obtained from this strain; coverage: fold-coverage of the overall read sequence with respect to the specific genome. Annotation: coding sequences (CDS) annotated to the specific overall sequences; no. of ribosomal (tRNA), transfer (tRNA) and transfer messenger (tmRNA) RNA CDS detected in the specific sequence. The three rRNAs listed correspond to single copies of the 5S, 16S and 23S rRNA genes, except for D16 and b04239, that possess extra and incomplete copies of the 16S and 23S rRNA genes, respectively. [file Table1.docx]

### Supplementary Table 1. Sequencing summary

Results from the overall bioinformatic analyses of the novel *C. acnes* sequences are shown in this table. Sequencing / Reads: overall no. of reads obtained from the specific *C. acnes* genome; bases: overall no. of bases read. Assembly / Length: length of the assembled genome in megabases; largest contig: size of the largest contig obtained from the genome of a specific strain, N50: median size of the contigs obtained from this strain; coverage: fold-coverage of the overall read sequence with respect to the specific genome. Annotation: coding sequences (CDS) annotated to the specific overall sequences; no. of ribosomal (tRNA), transfer (tRNA) and transfer messenger (tmRNA) RNA CDS detected in the specific sequence. The three rRNAs listed correspond to single copies of the 5S, 16S and 23S rRNA genes, except for D16 and b04239, that possess extra and incomplete copies of the 16S and 23S rRNA genes, respectively.

|  | sequencing | | assembly | | | | | annotation | | | |
| --- | --- | --- | --- | --- | --- | --- | --- | --- | --- | --- | --- |
| isolate | Reads  (mill.) | Bases  (Mb) | Length  (Mb) | Largest  contig (Kb) | GC (%) | N50 (Kb) | coverage | CDS | rRNA | tRNA | tmRNA |
| D13 | 2.22 | 347.79 | 2.48 | 893.59 | 60.05 | 467.39 | 278.31 | 2,295 | 3 | 46 | 2 |
| D14 | 2.76 | 361.56 | 2.48 | 893.64 | 60.05 | 434.88 | 288.14 | 2,301 | 3 | 46 | 2 |
| D15 | 1.94 | 332.65 | 2.48 | 893.62 | 60.06 | 471.62 | 264.74 | 2,298 | 3 | 46 | 2 |
| D16 | 2.23 | 365.18 | 2.48 | 893.61 | 60.02 | 720.30 | 292.34 | 2,295 | 4 | 46 | 2 |
| D17 | 2.08 | 331.86 | 2.53 | 732.71 | 60.14 | 277.74 | 258.89 | 2,353 | 3 | 46 | 2 |
| D18 | 2.30 | 366.65 | 2.48 | 893.34 | 60.05 | 722.05 | 292.33 | 2,291 | 3 | 46 | 2 |
| D19 | 1.90 | 255.40 | 2.48 | 893.32 | 60.05 | 469.15 | 202.63 | 2,292 | 3 | 46 | 2 |
| D20 | 2.51 | 353.13 | 2.48 | 893.31 | 60.04 | 722.07 | 281.49 | 2,311 | 3 | 46 | 2 |
| D21 | 0.45 | 77.73 | 2.49 | 494.48 | 60.05 | 275.98 | 61.42 | 2,310 | 3 | 46 | 1 |
| K1 | 1.41 | 156.45 | 2.49 | 347.70 | 60.01 | 201.66 | 122.92 | 2,306 | 3 | 46 | 2 |
| K10 | 1.10 | 121.20 | 2.48 | 347.69 | 60.04 | 201.60 | 95.17 | 2,306 | 3 | 46 | 2 |
| K11 | 0.77 | 95.23 | 2.53 | 302.04 | 60.17 | 145.60 | 73.81 | 2,367 | 3 | 46 | 2 |
| K12 | 0.79 | 89.76 | 2.48 | 732.76 | 60.04 | 165.25 | 70.72 | 2,303 | 3 | 46 | 2 |
| K2 | 1.94 | 237.71 | 2.54 | 434.90 | 60.07 | 271.79 | 184.13 | 2,371 | 3 | 46 | 2 |
| K5 | 2.44 | 278.44 | 2.54 | 454.70 | 60.10 | 342.96 | 214.99 | 2,351 | 3 | 46 | 2 |
| K7 | 1.70 | 195.38 | 2.54 | 372.94 | 60.05 | 183.66 | 148.81 | 2,374 | 3 | 46 | 2 |
| K8 | 1.94 | 200.75 | 2.54 | 508.04 | 60.06 | 252.60 | 153.78 | 2,352 | 3 | 48 | 1 |
| K9 | 0.95 | 103.82 | 2.48 | 385.34 | 60.03 | 252.08 | 80.77 | 2,302 | 3 | 46 | 2 |
| b02908 | 0.65 | 110.26 | 2.50 | 388.21 | 60.04 | 296.00 | 87.05 | 2,312 | 3 | 46 | 1 |
| b03014 | 1.45 | 148.30 | 2.54 | 384.93 | 60.02 | 187.77 | 112.81 | 2,356 | 3 | 47 | 2 |
| b03270 | 1.10 | 183.11 | 2.48 | 773.96 | 60.04 | 339.98 | 146.29 | 2,324 | 3 | 46 | 1 |
| b04239 | 0.50 | 59.17 | 2.56 | 276.38 | 60.01 | 176.24 | 44.55 | 2,369 | 4 | 47 | 2 |
| b04269 | 1.07 | 174.85 | 2.54 | 885.86 | 60.10 | 300.20 | 136.60 | 2,389 | 3 | 46 | 1 |
| b04543 | 1.38 | 210.71 | 2.55 | 378.81 | 60.03 | 301.75 | 163.18 | 2,357 | 3 | 47 | 2 |
| b04764 | 0.87 | 133.93 | 2.48 | 781.30 | 60.05 | 343.46 | 106.89 | 2,298 | 3 | 46 | 2 |
| b05051 | 1.33 | 158.00 | 2.55 | 323.83 | 60.01 | 187.79 | 119.76 | 2,360 | 3 | 47 | 2 |
| b05914 | 0.73 | 76.06 | 2.55 | 249.86 | 60.03 | 145.94 | 57.75 | 2,354 | 3 | 47 | 2 |
| b05979 | 2.65 | 324.76 | 2.49 | 488.72 | 60.04 | 332.33 | 257.31 | 2,320 | 3 | 46 | 1 |
| v01026 | 0.52 | 66.42 | 2.55 | 272.88 | 60.03 | 121.57 | 49.24 | 2,357 | 3 | 47 | 2 |
| v03925 | 0.57 | 71.08 | 2.49 | 494.42 | 60.02 | 175.02 | 55.75 | 2,312 | 3 | 46 | 1 |
| v03992 | 1.21 | 135.98 | 2.53 | 400.79 | 60.04 | 213.68 | 105.21 | 2,350 | 3 | 47 | 2 |
| v04016 | 1.48 | 187.09 | 2.49 | 434.88 | 60.03 | 253.00 | 148.19 | 2,299 | 3 | 46 | 2 |
| v04083 | 1.42 | 170.64 | 2.53 | 643.94 | 60.10 | 252.12 | 131.72 | 2,356 | 3 | 46 | 2 |
| v04290 | 1.05 | 131.63 | 2.48 | 575.29 | 60.05 | 438.37 | 104.15 | 2,313 | 3 | 46 | 1 |
| v04857 | 1.23 | 141.83 | 2.49 | 883.79 | 60.04 | 269.84 | 111.00 | 2,306 | 3 | 46 | 1 |
| v04915 | 1.79 | 226.57 | 2.55 | 384.94 | 60.03 | 218.94 | 174.71 | 2,348 | 3 | 47 | 2 |
| v06486 | 1.27 | 178.40 | 2.54 | 508.07 | 60.06 | 229.49 | 139.03 | 2,353 | 3 | 48 | 1 |
| v07195 | 1.10 | 156.19 | 2.54 | 781.24 | 60.06 | 252.92 | 120.88 | 2,352 | 4 | 49 | 1 |
| v07786 | 1.42 | 193.79 | 2.54 | 786.11 | 60.11 | 277.72 | 150.70 | 2,363 | 3 | 46 | 2 |
| v07956 | 1.63 | 252.16 | 2.50 | 871.01 | 60.04 | 388.22 | 199.32 | 2,318 | 3 | 46 | 1 |
| v08288 | 1.41 | 193.96 | 2.54 | 528.04 | 60.03 | 270.69 | 150.82 | 2,351 | 3 | 47 | 2 |
| v08359 | 0.90 | 144.06 | 2.49 | 883.79 | 60.04 | 369.42 | 114.39 | 2,313 | 3 | 46 | 1 |
| v12811 | 1.02 | 114.32 | 2.49 | 507.84 | 60.10 | 250.79 | 89.52 | 2,311 | 3 | 46 | 2 |
| v14082 | 1.71 | 195.97 | 2.55 | 443.20 | 60.03 | 252.95 | 150.65 | 2,352 | 3 | 47 | 2 |
