# Supplementary material for: Whole genome sequencing distinguishes skin colonizing from infection-associated Cutibacterium acnes isolates
Source: Front Cell Infect Microbiol. 2024 Oct 24;14:1433783. doi: 10.3389/fcimb.2024.1433783 (PMC11540793; doi:10.3389/fcimb.2024.1433783)
Supplement: Supplementary Table 2 — Complete C. acnes genomes from RefSeq. Complete C. acnes genomes included in the study. The respective sizes, strain names and references are indicated for each NCBI RefSeq accession. Genomes corresponding to the type strain (NZ_CP023676 and NZ_CP044255) are indicated in bold. The type strain is stored in different databases and repositories and therefore has several unique descriptors. Isolation source is indicated if retrievable from primary and secondary references. 1 Search in primary and secondary reference unfortunately did not allow easy grouping into acne and healthy individual isolates since tables showing isolate names and isolation sources for these isolates are missing in those publications. [file Table2.docx]

### Supplementary Table 2. Complete *C. acnes* genomes from RefSeq

Complete *C. acnes* genomes included in the study. The respective sizes, strain names and references are indicated for each NCBI RefSeq accession. Genomes corresponding to the type strain (NZ_CP023676 and NZ_CP044255) are indicated in bold. The type strain is stored in different databases and repositories and therefore has several unique descriptors. Isolation source is indicated if retrievable from primary and secondary references. ^1^ Search in primary and secondary reference unfortunately did not allow easy grouping into acne and healthy individual isolates since tables showing isolate names and isolation sources for these isolates are missing in those publications.

| Accession | Size (Mb) | Strain (ID, isolation source if known) | SLST type | traditional  phylotype | Reference |
| --- | --- | --- | --- | --- | --- |
| NC_006085 | 2.56 | KPA171202, DSM 16374, contamination of an aerobic culture | H2 | IB | 10.1126/science.1100330 |
| NC_014039 | 2.50 | SK137 | C1 | IA_1_ | Unpublished |
| NC_016511 | 2.50 | TypeIA2 P.acn31, aqueous humour | F4 | IA_2_ | 10.1128/JB.06758-11 |
| NC_016512 | 2.52 | TypeIA2 P.acn17, corneal scrape | F5 | IA_2_ | 10.1128/JB.06758-11 |
| NC_016516 | 2.49 | TypeIA2 P.acn33, no information in publication found | F1 | IA_2_ | 10.1128/JB.06758-11 |
| NC_017534 | 2.49 | 266,  pleuropulmonary infection | A1 | IA_1_ | 10.1371/journal.pone.0021581 |
| NC_017535 | 2.56 | 6609, female skin, no record of acne | H1 | IB | 10.1128/JB.05372-11 |
| NC_017550 | 2.49 | subsp. defendens ATCC 11828, subcutaneous abscess | K9 | II | 10.1128/JB.06388-11 |
| NC_018707 | 2.52 | C1 | A5 | IA_1_ | 10.1128/genomeA.00016-12 |
| NC_021085 | 2.49 | HL096PA1 = NCBI GenBank designated reference genome, clinsing pore strip sample, microcomidones associated | C1 | IA_1_ | 10.1038/jid.2013.21 |
| NZ_AP019664 | 2.49 | TP-CU389, acne isolate | F1 | IA_2_ | 10.1128/AAC.01810-19 |
| NZ_AP019723 | 2.49 | subsp. acnes NBRC 107605 | A1 | IA_1_ | Unpublished |
| NZ_AP022844 | 2.49 | SZ1 | A2 | IA_1_ | Unpublished |
| NZ_AP022845 | 2.50 | SZ2 | A2 | IA_1_ | Unpublished |
| NZ_CP006032 | 2.49 | hdn-1 | A1 | IA_1_ | Unpublished |
| NZ_CP012350 | 2.53 | PA_30_2_L1^1^ | D1 | IA_1_ | 10.1038/srep20662 |
| NZ_CP012351 | 2.56 | PA_21_1_L1^1^ | H1 | IB | 10.1038/srep20662 |
| NZ_CP012352 | 2.54 | PA_15_2_L1^1^ | A1 | IA_1_ | 10.1038/srep20662 |
| NZ_CP012353 | 2.47 | PA_12_1_R1^1^ | A1 | IA_1_ | 10.1038/srep20662 |
| NZ_CP012354 | 2.49 | PA_12_1_L1^1^ | A1 | IA_1_ | 10.1038/srep20662 |
| NZ_CP012355 | 2.49 | PA_15_1_R1^1^ | C1 | IA_1_ | 10.1038/srep20662 |
| NZ_CP012647 | 2.52 | KCOM 1861 | K2 | II | Unpublished |
| **NZ_CP023676** | 2.50 | ATCC 6919 | A1 | IA_1_ | Unpublished |
| **NZ_CP025934** | 2.50 | DSM 1897 | A1 | IA_1_ | Unpublished |
| NZ_CP025935 | 2.56 | KPA171202 | H2 | IB | Unpublished |
| NZ_CP031442 | 2.56 | KCOM 1315 | H1 | IB | Unpublished |
| NZ_CP033718 | 2.50 | FDAARGOS_577 | C1 | IA_1_ | Unpublished |
| NZ_CP033842 | 2.49 | FDAARGOS_503 | A1 | IA_1_ | Unpublished |
| **NZ_CP044255** | 2.49 | ATCC 6919 | A1 | IA_1_ | Unpublished |
| NZ_CP084017 | 2.56 | subsp. acnes NBRC 113869 | H15 | IB | Unpublished |
