# Supplementary material for: Whole genome sequencing distinguishes skin colonizing from infection-associated Cutibacterium acnes isolates
Source: Front Cell Infect Microbiol. 2024 Oct 24;14:1433783. doi: 10.3389/fcimb.2024.1433783 (PMC11540793; doi:10.3389/fcimb.2024.1433783)
Supplement: Supplementary Table 3 — Phage predictions. Prophages were identified with PHASTER (Arndt et al., 2016). “Most common” stands for the most commonly found phage by PHASTER, and “Accession” is the NCBI genome accession for this most commonly found phage. [file Table3.docx]

### Supplementary Table 3. Phage predictions

Prophages were identified with PHASTER (Arndt et al. 2016). “Most common” stands for the most commonly found phage by PHASTER, and “Accession” is the NCBI genome accession for this most commonly found phage.

| Isolate | Contig | Start | End | Most common | Accession |
| --- | --- | --- | --- | --- | --- |
| K8 | N5_L232072_C19.016288 | 154774 | 167761 | Lederberg | NC_048790 |
| b03014 | N13_L84961_C13.628852 | 7522 | 20509 | Lederberg | NC_048790 |
| b04239 | N1_L276377_C6.809180 | 97450 | 110437 | Lederberg | NC_048790 |
| b04543 | N5_L270165_C34.602608 | 63917 | 76904 | Adelaide | NC_048791 |
| b05051 | N7_L174925_C18.318894 | 64453 | 77440 | Adelaide | NC_048791 |
| b05914 | N10_L95487_C7.631481 | 74979 | 87966 | Lederberg | NC_048790 |
| v01026 | N11_L92364_C7.814424 | 64427 | 77415 | Lederberg | NC_048790 |
| v04915 | N3_L270875_C29.887984 | 193274 | 206261 | Adelaide | NC_048791 |
| v06486 | N9_L105109_C27.281772 | 27806 | 40793 | Adelaide | NC_048791 |
| v07195 | N9_L105107_C24.059326 | 27806 | 40793 | Lederberg | NC_048790 |
| v08288 | N4_L270686_C27.628340 | 193273 | 206260 | Adelaide | NC_048791 |
| v08526 | N12_L88258_C21.017485 | 64453 | 77440 | Lederberg | NC_048790 |
| v14082 | N7_L174888_C23.067750 | 97448 | 110436 | Adelaide | NC_048791 |
