# Supplementary material for: Whole genome sequencing distinguishes skin colonizing from infection-associated Cutibacterium acnes isolates
Source: Front Cell Infect Microbiol. 2024 Oct 24;14:1433783. doi: 10.3389/fcimb.2024.1433783 (PMC11540793; doi:10.3389/fcimb.2024.1433783)
Supplement: Supplementary Table 4 — Recombination sites. Recombination sites were identified with Gibbons upon comparison of the complete C. acnes genomes versus the NCBI GenBank designated reference genome HL096PA1. “Start” and “end” indicate the genomic coordinates with respect to the HL096PA1 genome (as well as their annotations in “gene” and “description”), while “value” is the density (SNPs per segment) to the corresponding genome position. Due to use of genomic coordinates instead of whole gene segments, the alr, coxB, erpA, and fumC genes appear more than once, i.e. these genes entail more than one recombination site. [file Table4.docx]

### Supplementary Table 4. Recombination sites

Recombination sites were identified with Gibbons upon comparison of the complete *C. acnes* genomes versus the NCBI GenBank designated reference genome HL096PA1. “Start” and “end” indicate the genomic coordinates with respect to the HL096PA1 genome (as well as their annotations in “gene” and “description”), while “value” is the density (SNPs per segment) to the corresponding genome position. Due to use of genomic coordinates instead of whole gene segments, the alr, coxB, erpA, and fumC genes appear more than once, i.e. these genes entail more than one recombination site.

| start | end | value | gene | description |
| --- | --- | --- | --- | --- |
| 435044 | 455170 | 873 | eda | bifunctional phosphogluconate aldolase |
| 435044 | 455170 | 873 | rlmB | 23S rRNA (guanosine(2251)-2'-O)-methyltransferase RlmB |
| 435044 | 455170 | 873 | amaP | alkaline shock response membrane anchor protein AmaP |
| 733675 | 743092 | 684 | fabG | 3-oxoacyl-ACP reductase FabG |
| 733675 | 743092 | 684 | fabI | enoyl-ACP reductase FabI |
| 974961 | 1001969 | 580 | serA | phosphoglycerate dehydrogenase |
| 974961 | 1001969 | 580 | aroA | 3-phosphoshikimate 1-carboxyvinyltransferase |
| 974961 | 1001969 | 580 | rsgA | ribosome small subunit-dependent GTPase A |
| 1472573 | 1499565 | 812 | uvrB | excinuclease ABC subunit UvrB |
| 1472573 | 1499565 | 812 | uvrA | excinuclease ABC subunit UvrA |
| 1572573 | 1589495 | 658 | ctaD | cytochrome c oxidase subunit I |
| 1572573 | 1589495 | 658 | erpA | iron-sulfur cluster insertion protein ErpA |
| 1573067 | 1589519 | 669 | erpA | iron-sulfur cluster insertion protein ErpA |
| 1572573 | 1589495 | 658 | coxB | cytochrome c oxidase subunit II |
| 1573067 | 1589519 | 669 | coxB | cytochrome c oxidase subunit II |
| 1573067 | 1589519 | 669 | ctaD | cytochrome c oxidase subunit I |
| 1911930 | 1951823 | 575 | glmS | glutamine--fructose-6-phosphate transaminase |
| 1911930 | 1951823 | 575 | rpsI | 30S ribosomal protein S9 |
| 1911930 | 1951823 | 575 | rplM | 50S ribosomal protein L13 |
| 1911930 | 1951823 | 575 | trmB | tRNA (guanosine(46)-N7)-methyltransferase TrmB |
| 1911930 | 1951823 | 575 | truA | tRNA pseudouridine(38-40) synthase TruA |
| 1911930 | 1951823 | 575 | fabG | 3-oxoacyl-ACP reductase FabG |
| 1911930 | 1951823 | 575 | rplQ | 50S ribosomal protein L17 |
| 1911930 | 1951823 | 575 | rpsD | 30S ribosomal protein S4 |
| 1911930 | 1951823 | 575 | rpsK | 30S ribosomal protein S11 |
| 1911930 | 1951823 | 575 | rpsM | 30S ribosomal protein S13 |
| 1911930 | 1951823 | 575 | rpmJ | 50S ribosomal protein L36 |
| 1911930 | 1951823 | 575 | infA | translation initiation factor IF-1 |
| 1911930 | 1951823 | 575 | map | type I methionyl aminopeptidase |
| 1911930 | 1951823 | 575 | secY | preprotein translocase subunit SecY |
| 2403014 | 2431858 | 1598 | alr | alanine racemase |
| 2411501 | 2431858 | 1030 | alr | alanine racemase |
| 2403014 | 2431858 | 1598 | fumC | class II fumarate hydratase |
| 2411501 | 2431858 | 1030 | fumC | class II fumarate hydratase |

## 
