# Supplementary material for: Whole genome sequencing distinguishes skin colonizing from infection-associated Cutibacterium acnes isolates
Source: Front Cell Infect Microbiol. 2024 Oct 24;14:1433783. doi: 10.3389/fcimb.2024.1433783 (PMC11540793; doi:10.3389/fcimb.2024.1433783)
Supplement: Supplementary Table 5 — Biosynthetic gene clusters. Listed are genes pertaining to biosynthetic gene clusters identified with antiSMASH in the C. acnes NCBI GenBank designated reference genome HL096PA1, together with their annotations (gene locus, product, function). These genes map to either the LAP (Linear azoline- containing peptides) or RiPP (unspecified ribosomally synthesized and post-translationally modified peptide product)- like regions, that both produce post-translationally modified peptides with various biological activities. [file Table5.docx]

### Supplementary Table 5. Biosynthetic gene clusters

Listed are genes pertaining to biosynthetic gene clusters identified with antiSMASH in the *C. acnes* NCBI GenBank designated reference genome HL096PA1, together with their annotations (gene locus, product, function). These genes map to either the LAP (Linear azoline- containing peptides) or RiPP (unspecified ribosomally synthesised and post-translationally modified peptide product)- like regions, that both produce post-translationally modified peptides with various biological activities.

| Gene/locus | Product | Function | Region |
| --- | --- | --- | --- |
| PAGK_RS00840 | type II toxin-antitoxin system HicA family toxin | other | LAP |
| PAGK_RS00850 | single-stranded DNA-binding protein | other | LAP |
| PAGK_RS00855 | ParA family protein | other | LAP |
| PAGK_RS00860 | hypothetical protein | other | LAP |
| PAGK_RS00865 | hypothetical protein | other | LAP |
| PAGK_RS00870 | hypothetical protein | other | LAP |
| PAGK_RS12235 | hypothetical protein | other | LAP |
| PAGK_RS13995 | hypothetical protein | other | LAP |
| PAGK_RS00880 | hypothetical protein | other | LAP |
| PAGK_RS00885 | ParA family protein | other | LAP |
| PAGK_RS12240 | hypothetical protein | other | LAP |
| PAGK_RS00890 | CPBP glutamic-type intramembrane protease | biosynthetic-additional | LAP |
| PAGK_RS00895 | CPBP intramembrane glutamic endopeptidase | biosynthetic-additional | LAP |
| PAGK_RS00900 | YcaO-like family protein | biosynthetic | LAP |
| PAGK_RS00905 | hypothetical protein | other | LAP |
| PAGK_RS00910 | SagB/ThcOx family dehydrogenase | biosynthetic | LAP |
| PAGK_RS00915 | EXLDI protein | other | LAP |
| PAGK_RS00920 | ABC transporter ATP-binding protein | transport | LAP |
| PAGK_RS00925 | ABC transporter permease | transport | LAP |
| PAGK_RS00930 | hypothetical protein | other | LAP |
| PAGK_RS13585 | hypothetical protein | other | LAP |
| PAGK_RS13590 | hypothetical protein | other | LAP |
| PAGK_RS00945 | hypothetical protein | other | LAP |
| PAGK_RS00955 | hypothetical protein | other | LAP |
| PAGK_RS00960 | HNH endonuclease family protein | other | LAP |
| PAGK_RS00965 | ATP-dependent helicase | other | LAP |
| PAGK_RS00970 | ECF transporter S component | other | LAP |
| PAGK_RS06940 | maleylpyruvate isomerase family mycothiol-dependent enzyme | other | RiPP-like |
| PAGK_RS06945 | MBL fold metallo-hydrolase | biosynthetic-additional | RiPP-like |
| PAGK_RS06950 | TerC family protein | other | RiPP-like |
| uvrB | excinuclease ABC subunit UvrB | other | RiPP-like |
| PAGK_RS06960 | lactococcin 972 family bacteriocin | biosynthetic | RiPP-like |
| PAGK_RS13710 | hypothetical protein | other | RiPP-like |
| PAGK_RS06970 | hypothetical protein | other | RiPP-like |
| PAGK_RS06975 | hypothetical protein | other | RiPP-like |
| PAGK_RS14195 | ATP-binding cassette domain-containing protein | transport | RiPP-like |
| PAGK_RS12865 | hypothetical protein | other | RiPP-like |
| PAGK_RS06985 | ABC transporter substrate-binding protein | transport | RiPP-like |

### 
