# Supplementary material for: Whole genome sequencing distinguishes skin colonizing from infection-associated Cutibacterium acnes isolates
Source: Front Cell Infect Microbiol. 2024 Oct 24;14:1433783. doi: 10.3389/fcimb.2024.1433783 (PMC11540793; doi:10.3389/fcimb.2024.1433783)
Supplement: Supplementary Table 6 — Regions with high SNP density. Single nucleotide polymorphisms (SNPs) were identified with snippy (REF) upon comparison of the novel infection-associated isolates versus the complete C. acnes genomes versus the NCBI GenBank designated reference genome HL096PA1. “Start” and “end” indicate the genomic coordinates with respect to the HL096PA1 genome (as well as their annotations in “gene” and “description”), while “value” is the density (SNPs per 0.1 Kb) to the corresponding genome position. [file Table6.docx]

### Supplementary Table 6. Regions with high SNP density

Single nucleotide polymorphisms (SNPs) were identified with snippy (REF) upon comparison of the novel infection-associated isolates versus the complete *C. acnes* genomes versus the NCBI GenBank designated reference genome HL096PA1. “Start” and “end” indicate the genomic coordinates with respect to the HL096PA1 genome (as well as their annotations in “gene” and “description”), while “value” is the density (SNPs per 0.1 Kb) to the corresponding genome position.

| start | end | value | gene/locus | description |
| --- | --- | --- | --- | --- |
| 79300 | 79400 | 6 | PAGK_RS00355 | FtsX-like permease family protein |
| 255200 | 255300 | 6 | PAGK_RS01125 | FtsW/RodA/SpoVE family cell cycle protein |
| 478500 | 478600 | 6 | PAGK_RS02250 | cobyric acid synthase |
| 478500 | 478600 | 6 | CbiB | adenosylcobinamide-phosphate synthase CbiB (cobD) |
| 557300 | 557400 | 6 | PAGK_RS02615 | UTP--glucose-1-phosphate uridylyltransferase |
| 557400 | 557500 | 9 | PAGK_RS02615 | UTP--glucose-1-phosphate uridylyltransferase |
| 1468900 | 1469000 | 8 | uvrC | excinuclease ABC subunit UvrC |
| 1472900 | 1473000 | 6 | uvrA | excinuclease ABC subunit UvrA |
| 1473100 | 1473200 | 6 | uvrA | excinuclease ABC subunit UvrA |
| 1572000 | 1572100 | 7 | noncoding | noncoding |
| 1585800 | 1585900 | 12 | PAGK_RS07450 | cytochrome c oxidase subunit 4 |
| 1680200 | 1680300 | 6 | PAGK_RS07875 | zinc ABC transporter substrate-binding protein |
| 1680400 | 1680500 | 6 | PAGK_RS07875 | zinc ABC transporter substrate-binding protein |
| 1866800 | 1866900 | 7 | PAGK_RS08770 | DUF6350 family protein |
| 2491700 | 2491800 | 11 | PAGK_RS11720 | RNA-binding protein |

## 
